# Research Progress on Hypoglycemic Effects and Molecular Mechanisms of Flavonoids: A Review

**DOI:** 10.3390/antiox14040378

**Published:** 2025-03-22

**Authors:** Mengyi Liu, Chunlong Liu, Puba Zhaxi, Xiaohong Kou, Yazhou Liu, Zhaohui Xue

**Affiliations:** 1 School of Chemical Engineering and Technology, Tianjin University, Tianjin 300072, China; liumengyi@tju.edu.cn (M.L.); kouxiaohong@tju.edu.cn (X.K.); 2 Tianjin Longsheng Biotechnology Co., Ltd., Tianjin 300450, China; lianyue886@163.com; 3 Key Laboratory of Functional Food and Food Quality and Safety, Food and Drug Inspection and Research Institute of Tibet Autonomous Region, Lhasa 850099, China; pbzx58@126.com (P.Z.); liuyazhou202103@163.com (Y.L.)

**Keywords:** flavonoids, diabetes, hypoglycemic, target, signal pathway, synergism, gut microbiota

## Abstract

As a prevalent metabolic disorder, the increasing incidence of diabetes imposes a significant burden on global healthcare. Flavonoids in natural phytochemical products exhibit notable hypoglycemic properties, making them potential alternatives for diabetes treatment. This article summarizes the hypoglycemic properties of flavonoid subcategories studied in recent years, including flavones, isoflavones, flavonols, flavanols, and others. The relevant targets and signal pathways, such as α-amylase, α-glucosidase, insulin receptor substrate (IRS)/phosphatidylinositol 3-kinase (PI3K)/protein kinase B (AKT), PKR-like endoplasmic reticulum kinase (PERK)/eukaryotic initiation factor 2α (eIF2α)/activation transcription factor 4 (ATF4)/C/EBP homologous protein (CHOP), etc., are also elaborated. Additionally, flavonoids have also been demonstrated to modulate the gut microbiota and its metabolites. Through the aforementioned mechanisms, flavonoids mainly suppress carbohydrate metabolism and gluconeogenesis; facilitate glucose uptake, glycogenesis, and insulin secretion; and mitigate insulin resistance, oxidative stress, inflammation, etc. Notably, several studies have indicated that certain flavonoids displayed synergistic hypoglycemic effects. In conclusion, this article provides a comprehensive review of the hypoglycemic effects of the flavonoids investigated in recent years, aiming to offer theoretical insights for their further exploration.

## 1. Introduction

Diabetes mellitus (DM) is a chronic disease associated with metabolic disorders of the endocrine system, characterized by high concentrations of blood glucose. In accordance with the etiology, type 1 diabetes (T1DM) and type 2 diabetes (T2DM) are the most common clinical types [1]. T1DM is significantly characterized by the absolute inadequacy of insulin secretion owing to pancreatic β-cell damage and destruction [2], whereas T2DM, accounting for more than 90% of all diabetes cases, is indicated by a relative inadequacy of insulin secretion caused by pancreatic β-cell dysfunction, as well as the depletion of insulin capacity to modulate glucose metabolism (insulin resistance, IR) [3].

Identified by the World Health Organization in 2019 as one of the top 10 causes of death worldwide from 2000 to 2019, diabetes is a severe threat to global health. The morbidity of diabetes has been growing at an alarming rate along with the improvement in living standards and alterations in lifestyle. It has been predicted that the prevalence of diabetes among adults aged 20–79 years could rise to 12.2% in 2045, meaning that there would be 783.2 million people with diabetes throughout the world. This represents staggering growth compared to the 10.5% (536.6 million people) living with diabetes in 2021. With the rising prevalence, the economic burden of diabetes is also enormous. The total expenditure for diabetes in China was projected to rise from USD 250.2 to USD 460.4 billion from 2020 to 2030, with an annual growth rate of 6.32% [4]. It was estimated that the global health expenditures related to diabetes would also rise to USD 1054 billion by 2045 [5]. Noteworthily, the explosion of diabetes mellitus will impose a huge burden on the global medical system and economy, resulting in an urgent need for strategies able to efficiently ameliorate and treat diabetes, along with its comorbidities.

Currently, a cure for DM is not available. Among all treatment options, lifestyle intervention, e.g., a reasonable diet, regular exercise, smoking cessation, and so on, remains paramount [6]. Medications for diabetes encompass oral and injectable preparations. The frequently used hypoglycemic oral drugs, including metformin, glinides, rosiglitazone, sulfonylureas, thiazolidinediones, glucagon-like peptide-1 agonists, α-glucosidase inhibitors (acarbose), and so on, may eventuate certain side effects when used permanently, such as lactic acidosis, excessive hypoglycemia, weight gain, gastrointestinal adverse reactions, and exacerbation of the hepatic and renal burden [7].

Natural products, including tannins, organic acids, polyphenols, terpenes, flavonoids, etc., have been validated to target the circulatory system, intestine, pancreas, hepatic tissue, muscular tissue, adipose tissue, etc., as potential therapeutic substitutes for diabetes treatment [8,9]. Natural products are a rich source of hypoglycemic medication discovery which, because of their mild effects, low risk of contraindications or harmful effects, and low cost, can replace traditional drugs that may cause many side effects when taken for a long period of time. Natural products can also promote the high-value utilization of agricultural resources [9]. Among these, flavonoids are important polyphenols naturally found in plants, vegetables, and fruits as a type of secondary metabolite, with anti-inflammatory, anti-cancer, pro-cardiovascular, anti-microbial, anti- severe acute respiratory syndrome coronavirus 2 (SARS-CoV-2), and other biological activities. The chemical structures of flavonoids are diverse and modifiable, endowing them with significant potential for drug development. Moreover, flavonoids display abundant targets and pathways for lowering blood glucose, receiving significant attention in recent years [10].

This review gathers information from recent years on natural plant-derived flavonoids used in the treatment of conditions consistent with diabetes, summarizes the flavonoids mentioned and their molecular mechanisms, and emphasizes the synergistic hypoglycemic effects between the flavonoids. The literature search was conducted across several databases (Web of Science, PubMed, Scopus, Wiley, and Springer) from 2022 to date, using key search terms including diabetes, type 2 diabetes (T2DM), hypoglycemia, hyperglycemia, blood glucose, glycemic control, glycosylated hemoglobin (HbA1c), insulin resistance, flavonoids, flavone, isoflavone, flavonol, flavanone, flavanonol, chalcone, and anthocyanin.

## 2. Hypoglycemic Flavonoids Reported in In Vivo and In Vitro Studies

**Figure 1:**
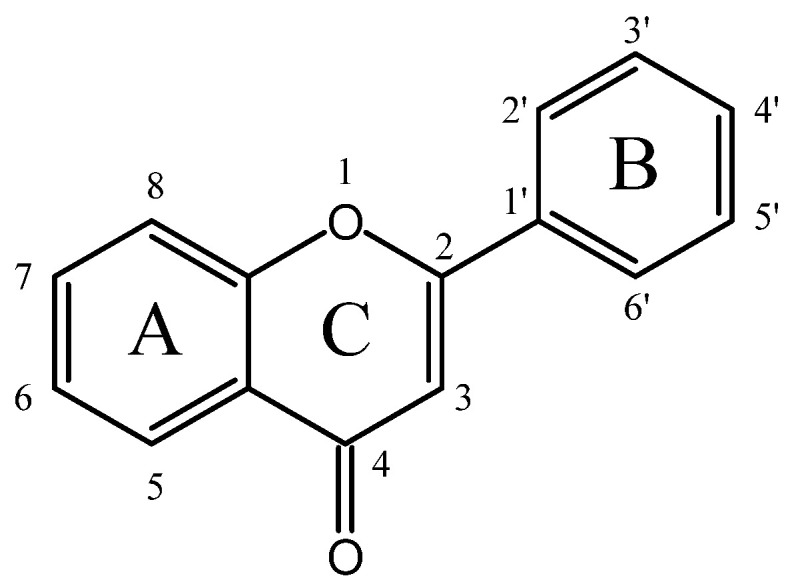
The basic skeleton of flavonoids.

Flavonoids generally possess a benzo-c-pyran (C6-C3-C6) carbon skeletal configuration, which is composed of a three-carbon heterocyclic pyran ring C connecting two benzene rings A and B, as shown in Figure 1 [11]. According to the substitutional pattern and oxidative degree of ring C, flavonoids can be categorized into different isoforms, such as flavones, isoflavones, flavonols, flavanols, flavanones, flavanonols, anthocyanins, etc., as listed in Table S1. In plants, flavonoids are mostly present in the form of C-glycosides and O-glycosides. In recent years, numerous in vitro, in vivo, and in silico studies have substantiated that different subclasses of flavonoids display strikingly hypoglycemic effects through various targets and signaling pathways (as indicated in Table S2), some of which are even more effective than clinical hypoglycemic agents, providing new insights for the treatment of diabetes.

### 2.1. Flavones

Differing from other flavonoids, flavones display a double bond between C2 and C3, with oxidation at C4 and no substitution at C3 in ring C.

Acacetin, mainly extracted from *Saussurea involucrate*, *Ziziphora clinopodioides Lam*, and *Robinia pseudoacacia*, reduced blood glucose levels and increased body weight in a streptozocin (STZ)-induced zebrafish model [12]; it also diminished fasting blood glucose (FBG) and relieved pancreas and liver injuries in high-fat diet (HFD) and STZ-treated diabetic rats [13].

Baicalin, present in the water extract of *Radix scutellariae*, could ameliorate hyperglycemia and glucose tolerance in T2DM mice [14]. In the research to evaluate the hypoglycemic effects of baicalein, baicalin (baicalein 7-O-glucuronide), and the hypoglycemic agent metformin, it was confirmed that baicalein is the most effective in diminishing glycation, α-glucosidase, and free radicals, while baicalin exhibited similar activities [15].

Eupafolin, present in *Artemisia princeps* and *Eremophila denticulate*, was demonstrated to reduce FBG, serum insulin, IR, and liver oxidative stress; normalize the lipid profile; and elevate the hepatic glycogen level in nicotinamide-STZ-induced diabetic rat models [16].

Luteolin, which was widely present in *Thespesia garckeana* F. Hoffm., *Rumex vescarius*, *Mentha arvensis*, and *Vernonia amygdalina*, significantly decreased blood glucose [17] and FBG levels, improved glucose tolerance and metabolic markers in plasma, slightly increased the number and area of β cells in islets [18], and alleviated palmitic acid (PA)-induced β cell apoptosis and the impairment of glucose-stimulated insulin secretion (GSIS) [19]. In vitro, luteolin was proven to inhibit the catalytic activity of α-amylase [20], α-glucosidase [18,21], and cyclooxygenase [22]. Luteolin-7-O-rutinoside was found to enhance GSIS and glucose uptake [23]. Lutexin (luteolin-8-glucoside), present in *Itea omeiensis*, competitively inhibited α-glucosidase activity [24].

Nobiletin, a polymethoxylated flavone present in orange and lemon peels, was proven to exert anti-glucotoxicity properties, such as reducing blood glucose and islet injuries and elevating insulin levels [25]. Tectochrysin (5-hydroxy-7-methoxyflavone), one of the major flavonoids in propolis, showed an insulin-mimetic effect, as it could potentiate glucose uptake while repressing liver gluconeogenesis, which led to decreased glucose levels and the enhanced insulin sensitivity [26]. Furthermore, 3,3′,4′,5,6,7,8-heptamethoxyflavone [27], a morusin abundant in *Morus alba* L. roots [28], improved glucose metabolism in diabetic mice, and genkwanin (separated from Vietnamese *Aquilaria crassna* leaves) [29], eupafolin [30], oroxylin A (derived from *Oroxylum indicum* Bentham ex Kurz) [31], swertisin (isolated from *Carex fraseriana*) [32], and schaftoside (present in sugarcane leaves) [33] exhibited inhibitory capacity against α-glucosidase or α-amylase.

### 2.2. Isoflavones

Isoflavones, also known as phytoestrogens, are abundant in legumes such as chickpeas, soy, beans, peanuts, and red clover [34].

Biochanin A, an O-methylated isoflavone, was demonstrated to markedly restore the abnormal levels of plasma glucose and plasma insulin, attenuate insulin resistance, reduce liver and pancreatic injuries, enhance glucose tolerance, and modulate the activities of glucose and glycogen metabolizing enzymes in the diabetic group [35].

Daidzein, a soy isoflavone, demonstrated the ability to reduce plasma glucose and body weight loss in STZ-induced diabetic rats [36].

Formononetin, a methoxy isoflavone, is present in many medicinal plants, such as *Astragalus membranaceus*, *Trifolium pratense* L., and *Pueraria lobata* (Willd.), as well as legumes like *Astragalus trimestris* L., exhibiting the capacity to downregulate serum glucose, insulin resistance, and fasting glucose levels and to upregulate serum insulin [37], while relieving islet injuries [38].

Puerarin (8-β-D-grapes pyranose-4,7-dihydroxy isoflavone), the main active component extracted from kudzu root, enhanced glucose tolerance and insulin sensitivity, mitigated liver dysfunction and hyperlipidemia, and attenuated oxidative stress and inflammation in HFD/STZ-induced mice and PA-treated murine hepatocytes [39].

Isoflavones from the *Apios americana* Medik. tuber displayed outstanding antioxidant tendencies and α-glucosidase inhibitory activity [40]. A study on *Crescentia cujete* L. indicated that among all the compounds isolated from the plant, isoflavone was the most effective inhibitor of α-glucosidase [41]. Seven isoflavones with α-glucosidase inhibitory activity were identified from black soybeans, and there was a negative correlation between the degree of glycosylation of isoflavone and the α-glucosidase inhibitory activity [42].

### 2.3. Flavonols

Flavonols have been identified in almost all plants, including gymnosperms, angiosperms, mosses, ferns, and liverworts. Compared with flavones, the core carbon skeleton of flavonols contains an additional alkoxy group at the C3 position of ring C.

Quercetin, as a crucial active ingredient in *Abelmoschus esculentus* (L.) Moench (okra) and *Euphorbia peplus*, was shown to be capable of alleviating body weight loss; reducing blood glucose, FBG, IR, hepatic and pancreatic injuries, oxidant stress, and inflammation; and increasing insulin levels and oral glucose tolerance in diabetic animal models [43,44,45]. In a study to investigate the impacts of quercetin, isoquercitrin, and rutin (a mixture of quercetin mono-glycoside and oligo-glycosides) on Institute of Cancer Research mice, quercetin glycosides, particularly isoquercitrin (but not aglycone), were eventually confirmed to suppress acute hyperglycemia [46]. Quercetin could also reduce postprandial hyperglycemia through directly interacting with starch and suppressing the activity of α-glucosidase [47,48].

Astragalin, identified from *Euphorbia peplus* and *Sauropus androgynus*, could increase hexokinase in diabetic rats [49] and showed a higher α-glucosidase inhibitory activity (half maximal inhibitory concentration, IC_50_) = 55.03 ± 0.61 μg/mL) than that of acarbose (162.96 ± 0.59 μg/mL) [50].

Kaempferol, as the main flavonoid of the extract of *Coreopsis tinctoria* and *Reseda lutea* L., alleviated body weight gain, FBG, serum glucose, and HbAc1 levels; ameliorated glucose tolerance, insulin resistance, the lipid profile, and liver injury; promoted glucose uptake in hyperglycemia mice compared with that in the control group [51,52,53]; and suppressed the activity of α-amylase and α-glycosidase [54], while heightening the hexokinase activity in HFD/STZ-induced diabetic rats [49].

Myricetin, isolated from *Annona cherimola* Miller, impeded monosaccharide absorption [55], minimized the serum glucose and insulin levels, and ameliorated the apoptosis of pancreatic tissues in a HFD/STZ-treated rat models [56]. The research evaluated the prospective hypoglycemic properties of three flavonol glycosides present in *Acacia mearnsii* leaves, i.e., myricitrin, myricetin-3-O-glucoside, and myricetin-3-O-arabinoside, which displayed α-glucosidase and α-amylase inhibitory activity, and the effect of myricitrin was the most prominent [10].

In addition, epimedin C, derived from *Epimedium* [57]; fisetin, which is present in cucumbers, onions, apples, and strawberries [58]; morin, richly expressed in the Moraceae family [59]; silibinin, which is the main component of silymarin extracted from *Cirsium japonicum* [60]; and isorhamnetin [61] were found to ameliorate blood glucose, glucose intolerance, insulin resistance, oxidative stress, and inflammation, or to elevate hepatic glycogen, i.e., GSIS. Narcissoside (isorhamnetin 3-rutinoside), present in various herbs such as *Anoectochilus roxburghii*, *Peucedanum aucheri Boiss.*, and *Colicodendron scabridum*, statically quenched α-glucosidase [62].

### 2.4. Flavanols

Unlike in the case of flavones, in flavanols, there are no double bonds between C2 and C3, nor is there oxidation at C4 of ring C; there is only an alkoxy group at the C3 position.

(-)-Epicatechin-6, isolated from Australian *Acacia saligna*, increased the uptake of glucose [63].

Epigallocatechin gallate (EGCG), an active substance obtained from Indian green tea extract, was proven to enhance the phosphorylation of insulin receptors and insulin sensitivity and promote glucose uptake in a dose-dependent manner [64]. EGCG also exhibited an inhibitory effect towards α-amylase, with an IC_50_ value of 0.548 ± 0.029 mg/mL [65].

Le et al. demonstrated that two galloylated flavanols, i.e., epi-catechin gallate and 3-O-galloyl-3,3′,5,5′,7-pentahydroxyflavan, extracted from the leaves of *Acer ginnala* Maxim., displayed inhibitory effects against α-glucosidase [66].

### 2.5. Flavanones

Flavanones have no double bond between the C2 and C3 of ring C, in contrast to the structure of flavones.

Bavachin isolated from the seeds and fruits of *Psoralea corylifolia* L. could ameliorate glucose homeostasis and insulin sensitivity [67]; pinocembrin (5,7-dihydroxyflavone) enhanced glucose consumption and improved insulin resistance [68].

Supplementation with hesperetin at a dose of 20 mg/kg b.wt. for 6 weeks remarkably diminished serum glucose and hepatic glycogen, augmented glucose tolerance through increasing pancreatic islet cells, and thus promoted insulin secretion in T1DM rats [69]. Hesperetin was proven to be a mixed inhibitor for human salivary α-amylase [70]. Neoeriocitrin (hesperetin 7-O-neohesperidoside) could enhance GSIS and decrease intracellular reactive oxygen species (ROS) [71]. Hesperidin (hesperetin 7-rutinoside), mainly present in citrus fruits, could uncompetitively inhibit α-glucosidase activity, with an IC_50_ value of 18.52 μM [72].

Naringenin (4,5,7-trihydroxy-flavanone), a flavonoid phytoestrogen, was determined to exert antidiabetic activity in different organs of STZ-induced mouse models, such as alleviating the apoptosis of islet β-cells and hepatocytes and regulating glucose metabolism in skeletal muscles [73]. In STZ/Nicotinamide-treated diabetic rat models, naringenin considerably improved IR, glucose, and insulin levels, increased glucose tolerance [74], and appreciably restored pancreatic β cell mass and insulin secretion at a dosage of 50 mg/kg for 2 weeks [75]. In vitro, naringenin was found to protect HepG2 cells from PA-induced insulin resistance [76].

Nigragenon O, separated from the stems of *Mours nigra*, decreased the glucose level in IR 3T3-L1 cells [77].

### 2.6. Flavanonols

For flavanonols, there is an alkoxy group at C3 and a carbon–oxygen double bond at C4 of ring C.

Dihydromyricetin (DHM), the main flavonoid component in *Ampelopsis grossedentata*, ameliorated hepatic steatosis and insulin resistance in both HFD-treated rats and PA-induced HepG2 cells [78]. Chen et al. carried out an in vivo study that confirmed that dihydromyricetin (250 mg/kg), administrated once a day by gavage for 12 weeks, significantly decreased FBG, triglycerides (TG), and HbA1c and increased fasting insulin (FINS) levels in STZ-treated diabetic mice [79].

Sanggenon C, isolated from the root bark of *Cortex Mori*, could improve glucose uptake and oxidative stress, as well as lower lipid levels, which attenuated insulin resistance in palmitic acid-induced HepG2 cells [80].

### 2.7. Biflavonoids

Biflavonoids, typically isolated from *Selaginella uncinate*, are naturally present dimeric compounds consisting of two flavonoid parts.

In a study of the potential antidiabetic activity of extracts from *Selaginella tamariscina* (P.Beauv.) Spring, two biflavonoids were proven to enhance glucose consumption in both normal and insulin-resistant HepG2 cells [81].

Amentoflavone, a C–C type representative flavonoid isolated from *Aletris spicata* and *Selaginella tamariscina*, could improve cell viability, insulin-stimulated glucose uptake, and GLUT4 expression, suppressing cell apoptosis and oxidative stress [82].

The two biflavones, amentoflavone and bilobetin, which differ only in the C8 position, with the former being hydroxyl and the latter methoxy, were both able to improve insulin resistance and aberrant insulin secretion [83]. Moreover, amentoflavone and hinokiflavone displayed stronger α-glucosidase inhibitory tendency than did acarbose [84].

Sadeghi et al. evaluated the inhibitory activities of 18 biflavonoids against α-glucosidase, ultimately confirming that strychnobiflavone achieved the best score with a binding energy of −8.85 kcal/mol, and it acted as an α-glucosidase inhibitor in a mix-competitive pattern [85].

### 2.8. Chalcones

Chalcones exhibit a core structure of two aromatic rings connected by a three-carbon unsaturated carbonyl group and are easily cycled, which makes them one of the most structurally diverse flavonoids [86].

Licochalcone A, identified in licorice (the rhizomes and roots of *Glycyrrhiza inflata* Bat., *Glycyrrhiza uralensis* Fisch., and *Glycyrrhiza glabra* L.), could enhance hepatic glycogenesis and inhibit gluconeogenesis in HFD-induced diabetic mice [87].

Neohesperidin, a bicyclic dihydrochalcone, regulated glucolipid metabolism and inflammatory responses and improved insulin resistance in diabetic zebrafish. Moreover, neohesperidin dihydrochalcone markedly relieved diabetes-exacerbated lipid accumulation in liver tissues [88]. Nothofagin, a type of dihydrochalcone extracted from *Aspalathus linearis* (Burman f.) R. Dahlgren, was found to exhibit anti-glycation activity [89].

Phlorizin, a dihydrochalcone present in *Lithocarpus litseifolius* (Hance) Chun, promoted glucose consumption, glucose uptake, and glycogen synthesis, as well as inhibited gluconeogenesis, oxidative stress, and lipid accumulation in free fatty acids (FFA)-induced insulin-resistant HepG2 cells [90].

### 2.9. Anthocyanins

Anthocyanins, generally available in fruits, vegetables, cereals, and beans, are the predominant water-soluble pigments in plants and are commonly linked to a sugar molecule, such as glucose, rhamnose, galactose, arabinose, and rutinose.

Cyanidin-3-O-glucoside (C3G), always present in fruits and vegetables with dark color, including blueberries and red bayberries, enhanced glucose consumption, glycogen synthesis, and insulin sensitivity [91]; reduced blood glucose [92]; improved pancreatic β cell secretory dysfunction and apoptosis [93] in diabetic db/db mice and IR hepatic cells; as well as exerted a significant inhibitory effect on α-glucosidase [94]. Cyanidin-3-O-β-glucoside-rich haskap (*Lonicera caerulea* L.) berry extract could ameliorate glucose tolerance and insulin sensitivity [95].

Delphinidin could maintain glucokinase activity [96]. Delphinidin-3-O-glucoside prominently enhanced glucose uptake in high glucose (HG)oleic acid (OA)-treated HepG2 cells [97]. Delphinidin-3-O-galactoside and delphinidin-3-O-glucoside exhibited the prominent scavenging ability of radicals, along with inhibitory activity against α-glucosidase [97], and reduced advanced glycation end-product (AGE) accumulation [98].

Malvidin-3-arabinoside exhibited an inhibitory capacity against AGE accumulation and the secretions of nitric oxide and pro-inflammatory cytokines in RAW264.7 cells [98]. Malvidin-3-O-galactoside could remove 2,2’-azino-bis(3-ethylbenzothiazoline-6-sulfonic acid radical cation (ABTS^+^), 1,1-diphenyl-2-picrylhydrazyl (DPPH), and oxygen free radicals; repress α-glucosidase activity; potentiate glucose uptake; and reduce total cholesterol (TC) and TG in HG-OA-evoked HepG2 cells [97].

Petunidin-3-O-galactoside and petunidin-3-O-glucoside enhanced glucose uptake in HepG2 cell models. It was confirmed that petunidin-type and malvidin-type anthocyanins induced more notable glucose uptake activity than did delphinidin-type anthocyanins. Moreover, the two flavonoids both significantly scavenged radicals and inhibited α-glucosidase activity [97].

### 2.10. Homoisoflavonoids

Homoisoflavonoids are a special subclass of flavonoids with rare occurrence in nature; they are uniquely characterized by having one more carbon (C9) than the regular flavonoids in a 16-carbon skeleton. They are classified into five groups based on their structures: sappanin-type, scillascillin-type, brazilin-type, caesalpin-type, and protosappanin-type.

(E)-5-hydroxy-7-methoxy-3-(2-hydroxybenzyl)-4-chromanone (HM-chromanone), isolated from *Portulaca oleracea* L., was able to reduce blood glucose and alleviate insulin resistance and inflammation in mice with endotoxin-induced insulin resistance [99].

HM-chromanone remarkedly restored impaired insulin pathways, reduced glucose production, and enhanced glycogen synthesis in ob/ob mice [100] and PA-induced HepG2 cells, respectively [101].

Brazilin-type homoisoflavonoids are commonly found in the heartwood of *Caesalpinia sappan*, *Caesalpinia echinate*, and *Haematoxylon campechianum*. Awotuya et al. examined nine brazilin-type homoisoflavonoids to determine their inhibition of protein tyrosine phosphatase 1B (PTP1B) [102].

## 3. Clinical Trials Regarding the Hypoglycemic Effect of Flavonoids

Although the hypoglycemic effect of flavonoids has been demonstrated in many animal models, in vitro experiments, and in silico modeling, the relevant clinical data are very limited. The trials to investigate individual flavonoids are as follows. Oral ingestion of hesperidin at a dose of 1 g/day for 12 weeks significantly lowered blood glucose (*p* < 0.05) in 129 T2DM patients with metabolic syndrome and neuropathy [103]. A 3-month double-blind, placebo-controlled trial involving 50 T2DM subjects showed that the administration of 500 mg/day of rutin significantly decreased FBG, insulin, HbA1c, homeostatic model assessment of insulin resistance (HOMA-IR) (*p* < 0.001), interleukin 6 (IL-6), malondialdehyde (MDA) (*p* < 0.05) levels and improved lipid profiles [104]. However, a randomized clinical trial involving 48 patients with T2DM validated that daily administration of 140 mg silymarin capsules for 12 weeks achieved no statistically significant difference in FBG (*p*  =  0.789), HbA1c (*p*  =  0.719), and lipid profiles compared to those of the control group. This may be due to the low dose, the short intervention period, the inclusion of fewer subjects, etc. [105].

Clinical trials of flavonoid mixtures include the following. A parallel, double-blind randomized controlled trial (RCT) (n = 45; age 63.4 ± 7.4 years; 64% male) revealed that 26 g (freeze-dried) of blueberries abundant in anthocyanins attenuated insulin (*p* < 0.01) and glucose levels (*p* < 0.001) induced by a high-fat/high-sugar meal after 24 h in individuals with metabolic syndrome [106]. A double-blind, randomized clinical trial (n = 30) showed that daily administration of 200 mg of Eriomin, containing 70% eriocitrin, 5% hesperidin, 4% naringin, 1% didymin, and 20% fiber material, for 12 weeks reduced blood glucose (−5%) by increasing glucagon-like peptide-1 (GLP-1) secretion (17%) and alleviated inflammation by decreasing IL-6 (−14%) and tumor necrosis factor-α (TNF-α) (−20%) in subjects with hyperglycemia (110–150 mg/dL) [107]. Another double-blind randomized controlled trial revealed that administration of 200 mg/day of Eriomin for 12 weeks decreased hyperglycemia (6%), increased GLP-1 blood levels (22%) (*p* < 0.05), and altered microbiota composition in prediabetic patients compared with the results for the placebo group [108]. A double blind, placebo-controlled crossover trial involving 25 healthy subjects showed that supplementation with the extract containing 320.4 mg of anthocyanins (mainly cyanidin and delphinidin) could mitigate high-fat diet-induced endotoxemia, reduce increases in plasma lipopolysaccharide (LPS) and LPS-binding protein, and lower blood glucose and triglycerides, but it had no significant effect on the increases in plasma insulin, GLP-1, GLP-2, or glucose-dependent insulinotropic polypeptide (GIP) [109]. A 5-week crossover study showed that dietary supplementation with elderberry juice containing pronounced anthocyanins significantly changed the gut microbiota and reduced blood glucose in 18 overweight or obese adults without chronic illnesses [110].

As can be seen from the above results, dietary supplementation with flavonoids exerts hypoglycemic actions by modulating blood glucose, insulin resistance, lipid profile, inflammation, oxidant stress, insulin signaling, endotoxemia, and gut microbiota. Additionally, comparative analysis shows that flavonoid mixtures are more effective than individual flavonoids at lower doses, and there are usually more glucose-lowering pathways involved due to the synergy of multiple flavonoids. However, it should be noted that in most of these clinical studies, the intervention period was 12 weeks, and the number of subjects was less than 50. Future clinical studies with longer intervention periods and larger numbers of participants are needed to verify the long-term hypoglycemic efficacy of flavonoids.

## 4. Molecular Mechanisms Underlying the Hypoglycemic Effects of Flavonoids

### 4.1. Targets

#### 4.1.1. Carbohydrate-Metabolizing Enzymes

Blood glucose originates from the digestion and absorption of exogenous foods and is produced through two endogenous pathways, namely gluconeogenesis and glycogenolysis, while it is metabolized through anaerobic oxidation (glycolysis) and aerobic oxidation, as well as the pentose phosphate pathway, with glycogen synthesis serving as a sink for glucose. Therefore, regulating the activities of enzymes involved in the above reactions, and thus balancing blood glucose production and consumption, is essential for maintaining blood glucose homeostasis. Numerous studies have substantiated that flavonoids can regulate the catalytic activities of enzymes involved in blood glucose metabolism, such as α-amylase, α-glucosidase, glucokinase, etc., thereby inhibiting blood glucose absorption, promoting blood glucose consumption, and alleviating hyperglycemia.

- α-amylase

Carbohydrates serve as one of the primary energy sources for the human body. In comparison to fats and proteins, consuming foods high in carbohydrates can lead to a significant increase in postprandial blood glucose, particularly in diabetic patients who have a relative or absolute deficiency of insulin. Consequently, modulating carbohydrate metabolism emerges as a crucial strategy for managing blood glucose levels.

As one of the carbohydrates, starch is hydrolyzed into oligosaccharides by salivary and pancreatic enzymes, which is the first step in digestion and absorption. As illustrated in Table 1, numerous flavonoids are found to modulate the activity of α-amylase, thus suppressing starch absorption and lowering blood sugar levels. The IC_50_ values indicated that the order of inhibitory effect of flavones was nepetin > scutellarein > apigenin > hispidulin [111], and the order of flavonols was kaempferol > quercetin > rutin [112].

Molecular docking results indicated that flavonoids spontaneously bind to α-amylase through non-covalent intermolecular interactions, including hydrogen bonds, hydrophobic interactions, and π-interactions [65,111,113], altering the structure and hydrophobic microenvironment of the enzyme and inhibiting α-amylase activity [112]. Specifically, the interaction between flavonoids and α-amylase diminished the α-helix content of the enzyme and modified its secondary structure, causing the enzyme to pronouncedly unfold, which hindered the substrate from binding to the active sites of the enzyme and influenced the microenvironment of Trp and Tyr residues in α-amylase, with a decreased polarity and an increased hydrophobicity around the residues [70,111]. Related studies have illustrated the structure–activity relationship between flavonoids and α-amylase, confirming the important role of hydroxyl groups. It was suggested that the number of OH groups in ring A and ring B and the inhibition of α-amylase presented a positive correlation; moreover, the OH groups at the A6, A7, B4’, B5’ position and the introduction of galloyl groups could enhance the inhibition of α-amylase [114,115]. That is because the hydroxyl groups located in these positions facilitate the formation of hydrogen bonds with the side chains of the amino acid residues of α-amylase. Moreover, ring C of flavones and flavonols is the 4-oxoflavonoid core, and the C2–C3 double bond is conjugated with the 4-ketone group on ring B, thus forming a highly conjugated π-system, resulting in better stability when interacting with the active site of the enzyme [116]. Noteworthily, the only difference between scutellarein, apigenin, and hispidulin was at the C6 position of ring A, where scutellarein was hydroxylated, and hispidulin was methoxylated compared to the results for apigenin [111]. However, the effects of rutin, quercetin, and kaempferol suggested that glycosylation at C3 on the C ring and hydroxylation at C3’ on the B ring attenuated the inhibitory effect [112].

Interestingly, Tonsic et al. discovered that although epicatechin, catechin, rutin, quercetin, and naringenin were in vitro inhibitors of α-amylase, only supplementation with 100 mg/kg of rutin inhibited starch absorption in mice, indicating that further in vivo studies are needed to verify the effect of α-amylase inhibitors on starch absorption in vivo [117].

- α-glucosidase

Normally, the oligosaccharides are further hydrolyzed into glucose by α-glucosidase in the small intestine. Therefore, restraining the activity of α-glucosidase also plays a pivotal role in restraining carbohydrate decomposition and controlling postprandial hyperglycemia [118]. A multitude of flavonoids have been proven to exert inhibitory effects on the catalytic activity of α-glucosidase, such as quercimeritrin [115], narcissoside [62], cyanidin-3-O-glucoside [94], etc. (Table 2). Some flavonoids are even more effective in vitro than are clinical α-glucosidase inhibitors (acarbose), such as scutellarein, nepetin, apigenin, hispidulin [118], and astragalin [50], and they could also synergistically inhibit α-glucosidase with acarbose [119]. According to the IC_50_ values, the order of inhibitory effect of flavonoids reported was scutellarein > nepetin > apigenin > hesperidin > hispidulin > luteolin.

Molecular docking results indicated that flavonoids usually interacted with the amino acid residues in α-glucosidase via the van der Waals force, hydrogen bonds, hydrophobic interactions and π-interactions, and electrostatic force [24,31,115,118,119] which certainly altered the conformation, secondary structure, and microenvironment of α-glucosidase, and then influenced its functionality [118]. As an illustration, the combination with narcissoside loosened and expanded the protein skeleton of α-glucosidase, declined the α-helix, and disrupted the helical structure of the peptide chain and peptide chain elongation [62]. Likewise, cyanidin-3-O-glucoside decreased the α-helix and β-angle and increased β-folding and irregular helix formation [94]. In addition, flavonoids, such as narcissoside [62], luteolin [21], oroxylin A [31], hinokiflavone, amentoflavone [84], and quercimeritrin [115] generally altered the microenvironment around tyrosine and tryptophan of α-glucosidase, enhancing its hydrophobicity.

Numerous studies implied that the augmentation of hydroxyl groups could strengthen the inhibitory activity of flavonoids against α-glucosidase since they participate in the interaction with the enzyme [21,84,118]. Flavonoids with two catechol groups in rings A and B, together with an OH group in the C3 position, display high inhibitory activity. The hydroxyl group at the A5, A7, A8, B3’, B4’, and C6 position of the flavonoids increased the inhibitory activity of α-glucosidase, while the methoxylation of the C3, C6, B3’, and B4’corresponding sites decreased the inhibitory activity [62,120]. Moreover, it was revealed that the glycosylation at the A7 position was more effective at inhibiting α-glucosidase than the hydroxylation at the A7 position or the glycosylation at the C3 position, while the hydroxylation at C3 is more favorable than the glycosylation at this position. Galactoside in the C3 position was more favorable than the presence of rhamnose and arabinofuranoside. The OH groups at the B4’ and B5’ positions were beneficial to the inhibition of α-glucosidase [115].

- Gluconeogenic enzymes

Gluconeogenic enzymes catalyze the conversion of various non-glucose substances into glucose or glycogen, including glucose-6-phosphatase (G6Pase), which hydrolyzes glucose-6-phosphate and stimulates glucose release from the tissues; phosphoenolpyruvate carboxykinase (PEPCK), the key enzyme that catalyzes the first step of gluconeogenesis; and fructose-1,6-bisphosphatase, which maintains glucose homeostasis in the liver and kidney. In DM patients, the activity of gluconeogenic enzymes usually increases due to impaired insulin secretion or action. However, this phenomenon can be reversed by flavonoids.

Treatments with hesperetin increased the activity of glycogen phosphorylase and decreased glucose-6-phosphatase activity, resulting in a decrease in hepatic glucose production, which plays an important role in the control of blood glucose levels in diabetic rats [69]. Dihydromyricetin relieved hepatic insulin resistance through the inhibition of hepatic gluconeogenesis and the enhancement of glucose uptake and hepatic glycogen content via decreasing the expression of G6Pase and PEPCK [78]. Biochanin A restored the downregulation of the glycolytic enzyme (hexokinase) and the upregulation of gluconeogenic enzymes (glucose-6-phosphatase and fructose-1,6-bisphosphatase) in diabetic rats [35]. Myricetin significantly inhibited G6Pase and PEPCK [56].

- Other enzymes

Glycogen metabolic enzymes contain glycogen synthase (inactive when phosphorylated) and glycogen phosphorylase. Deficiency in insulin and resistance to insulin action cause the stimulation of glycogenolytic and gluconeogenic processes [69]. Biochanin A was able to increase glycogen in the liver and muscles and decrease glycogen in the kidneys via the regulation of glycogen synthase and glycogen phosphorylase activities [35].

**Figure 2:**
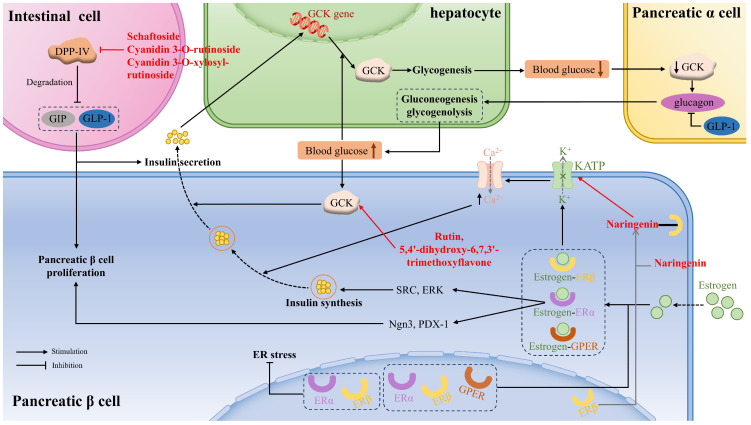
The mechanism of flavonoids in regulating glucose metabolism, pancreatic β cell proliferation, and function via glucokinase (GCK), dipeptidyl peptidase IV (DPP-IV), estrogen receptors (ER), and GLP-1 between liver cells, pancreatic cells, and intestinal L cells. GPER: G protein-coupled ER; SRC: non-receptor tyrosine-protein kinase; ERK: extracellular signal-regulated kinase; Ngn3: neurogenin3; PDX-1: pancreatic and duodenal homeobox 1.

Glucokinase (GCK), also known as hexokinase, is often referred to as a glucose sensor, mainly occurring in liver cells and pancreatic cells, that plays a crucial role in whole-body glucose homeostasis. That means that when the blood glucose level rises to a certain threshold, GCK in pancreatic β cells is activated to stimulate insulin secretion. If blood glucose continues to rise, GCK in the liver cells will be activated to facilitate hepatic glycogen production. Moreover, GCK is a key determinant in glucose sensing within α-cells to regulate glucagon secretion, gluconeogenesis, and hepatic glycogenolysis. Flavonoids acting as small-molecule GCK activators not only reduce fasting and basal blood glucose levels but also improve glucose tolerance. Rutin and 5,4′-dihydroxy-6,7,3′-trimethoxyflavone showed appreciable binding interactions with the allosteric site residues of the human GCK, thus activating the enzyme [121] (Figure 2).

Moreover, glucokinase regulatory protein (GKRP) regulates GCK activity by establishing the GCK–GKRP complex, causing GCK to be inactive. It was demonstrated that some anthocyanidins showed lower binding energy to GKRP than did fructose-1-phosphate (F1P) (a well-known inhibitor of the GKRP–GCK complex), especially delphinidin, cyanidin, and pelargonidin, thus preventing the formation of GKRP–GCK and maintaining glucokinase activity [96].

#### 4.1.2. Diabetic Kinome

The diabetic kinome is composed of protein kinases known to play a key role in modulating cell processes engaged in diabetes progression. For instance, the kinases responsible for the modulation of β cell proliferation and function, such as inhibiting dual specificity tyrosine phosphorylation-regulated kinase 1 A (DYRK1A), DPP-IV, and death-associated protein kinase-related apoptosis-inducing kinase-2 (Drak2). Additionally, the mechanistictarget of rapamycin (mTOR) and PTP1B plays a central role in insulin signaling.

DYRK1A has been shown to promote both insulin secretion and β cell proliferation. Five flavones and three aglycones (quercetin, baicalein, and herbacetin) were demonstrated to dose-dependently decrease DYRK1A activity in vitro, improve cell viability, and promote β-cells proliferation and insulin production. Mechanistically, the flavones were found to bind to the ATP-binding pocket of DYRK1A owing to the OH groups [122].

DPP-IV, a serine protease, is responsible for degrading GIP and GLP-1, which are peptide hormones secreted by intestinal cells to promote β-cell proliferation and insulin secretion. Some flavonoids can affirmatively inhibit DPP-IV activity and then ameliorate insulin-mediated glucose metabolism. The catalytic triad consisting of Ser630, Asn710, and His740 within the S1 pocket significantly affects DPP-IV activity. Schaftoside, a flavone that is present in sugarcane leaves, primarily repressed DPP-IV activity by forming hydrogen bonds, as well as carbon–hydrogen bonds, with the residues (Arg125, Glu205, Ser209, Ser630, Tyr666) [33]. Cyanidin 3-O-rutinoside, cyanidin 3-O-xylosyl-rutinoside, and malvin were affirmed to combine with amino acid residues in the active pocket of DPP-IV via salt bridge formation, π–π stackings, and hydrogen bonds [113] (Figure 2).

Drak2 plays a crucial role in modulating β cell survival. Luteolin potently inhibited Drak2 activity, with an IC_50_ of 346.7 ± 30.0 nM, as well as the expression of Drak2, thereby alleviating PA-induced β cell apoptosis and GSIS impairment owing to the promotion of autophagy and the mitigation of oxidative stress in INS-1E cells [19].

mTOR is a serine and threonine protein kinase with a specific role in insulin resistance. mTORC1 negatively regulates insulin signaling through a (p70S6) S6K-dependent feedback loop, which diminishes the interaction of insulin receptors and their downstream effectors. Licochalcone A enhanced glucose tolerance, insulin-induced glucose uptake, and insulin sensitivity through repressing mTORC1, which attenuated the negative impact of S6K on insulin signaling in diabetic mice [87].

PTP1B is a crucial tyrosine phosphatase involved in the regulation of insulin receptors [102] and is expressed in most cells in the body; however, it is overexpressed under diabetic conditions, which exacerbates insulin resistance. Consequently, PTB1B inhibitor has the potential to stimulate insulin-regulated glucose uptake, enhance insulin sensitivity, and mitigate insulin resistance, and it is regarded as a promising therapeutic agent for the treatment of diabetes [123]. Numerous flavonoids have been demonstrated to be potent PTB1B inhibitors. C3G was proven to suppress the expression of PTP1B, thereby strengthening the sensitivity of hepatic cells to insulin [91]. Among all identified O-methylated flavonoids from *Eremophila clarkei*, hispidulin showed the strongest inhibition against PTP1B, with an IC_50_ value of 24.8 ± 1.9 μM [124]. Cyanidin 3-(p-coumaroyl)-diglucoside-5-glucoside displayed a high binding affinity of −15.272 kcal/mol for PTP1B [113]. Epigallocatechin gallate (EGCG) and ECG could inhibit PTP1B and low molecular weight (LMW)-PTP. ECGG was proven to suppress PTP1B non-competitively by binding to multiple active sites of PTP1B via hydrogen bonds and hydrophobic interactions [64]. Baicalin, baicalein, chrysin, trihydroxy-dimethoxyflavone, salvigenin, and norwogonin displayed an outstanding binding affinity towards PTP1B through π–π and π–alkyl interactions [125].

#### 4.1.3. Estrogen Receptors

Estrogen exerts its function by binding to the estrogen receptor (ER), including ERα, Erβ, and GPER, which can all be expressed in pancreatic β cells, important estrogen target organs. The activation of estrogen receptors is conducive to β cell proliferation and function, thereby improving systemic glucose metabolism. Moreover, the activation of ERs increases the secretion of GLP-1.

The activation of ERα in β cells promotes cell proliferation or regeneration through activating Ngn3 or PDX-1, increases proinsulin gene expression, followed by insulin synthesis, through extracellular ERα signaling to SRC and ERK. The activation of ERβ leads to the membrane atrial natriuretic peptide receptor-dependent reduction of KATP channel activity, leading to the opening of the voltage-dependent Ca^2+^ channel. The increased intracellular Ca^2+^ concentration promotes the fusion of insulin vesicles with the cell membrane and the subsequent release of insulin. GPER is also involved in GSIS in the β cells, which is related to the EGFR, ERK, and the AKT/mTOR/glucose transporter 2 (GLUT2) signaling pathways [75] (Figure 2).

Naringenin, a flavonoid phytoestrogen with potential estrogen-like effects, was found to specifically activate ERβ without affecting other ER isoforms, thus appreciably improving islet morphology, augmenting the percentage of β cell area in the pancreas, and elevating insulin secretion, which improved blood glucose and glucose tolerance in diabetic rat models. Further study revealed that naringenin directly bound to ERβ, activated the membrane atrial natriuretic peptide receptor, and subsequently decreased KATP channel activity, which enhanced calcium ion influx and promoted insulin release. Moreover, chronic administration of naringenin markedly upregulated the expression of genes related to β-cell function [75]. Silibinin was proven to reduce PA-caused GLUTag cell ER stress through the regulation of ERα and ERβ, improving GLP-1 secretion [126].

#### 4.1.4. Glucose Transporter

Glucose transport proteins comprise sodium-dependent glucose transporters (SGLT) and facilitative GLUT. SGLT is a group of transmembrane proteins, including SGLT1 and SGLT2, whose main function is to mediate the transport of glucose and other monosaccharides on the cell membrane utilizing a gradient of sodium ions. SGLT plays an important role in the absorption and transport of glucose, especially in the intestine and kidneys: SGLT1, which is mainly expressed in the small intestine and kidneys, transports glucose from the intestinal lumen into intestinal epithelial cells, where it then enters the bloodstream through cytoplasmic transporters like GLUT, as well as prevents the loss of glucose in urine, while SGLT2 is predominantly expressed in the kidneys and acts as a main target responsible for transporting glucose from glomerular filtrate and glucose reabsorption. Thereupon, inhibiting SGLT is regarded as a promising strategy for controlling blood glucose levels, and many flavonoids have this effect.

For instance, molecular docking revealed that the ΔG value of myricetin and SGLT1 was −7.89 kcal·mol^−1^, while that of canagliflozin (a clinical inhibitor of SGLT1) was −10.08 kcal·mol^−1^, indicating a similar binding ability to SGLT1. Additionally, myricetin interacted with some aminoacidic residues (Asn78, His83, Glu102, Thr287, Tyr290, Trp291, Gln457) of SGLT1, playing a vital role in glucose transport and thus, impeding SGLT1-mediated glucose absorption [55]. Naringenin could occupy the glucose binding sites of proton-dependent glucose transporters via strong hydrophobic interactions, especially in the protonation state, limiting the glucose transport by SGLT [127].

GLUT transports monosaccharides through a diffusion gradient. As one of the GLUT isoforms, GLUT1 exists in a wide range of tissues to maintain the basal glucose supply. GLUT2, the most abundant GLUT isoform in hepatocytes, is accountable for most of the glucose uptake. GLU2 and GLUT3 participate in pancreatic insulin secretion. GLUT4 presents a pivotal role in regulating systemic glucose homeostasis and insulin-stimulated glucose transport. A number of documents elucidated that flavonoids can ameliorate glucose metabolism by enhancing the expression of GLUT. For example, liquiritigenin, naringenin, apigenin, kaempferol, and glabridin, isolated from the dichloromethane soluble fraction of the stem extract of *Wendlandia tinctoria* var. grandis (Roxb.) DC., exerted excellent hypoglycemic activities via combining with GLUT3 [128]. Myricetin significantly enhanced the expression of insulin receptors and GLUT4 [56]. Isoquercitrin, rutin [46], and isorhamnetin [129] promoted the translocation of GLUT4 to the plasma membrane.

#### 4.1.5. The Human Islet Amyloid Polypeptide (hIAPP)

hIAPP, a peptide composed of 37 amino acids, is highly amyloidogenic and is synthesized in pancreatic β-cells, where it is co-secreted with insulin. The soluble and naturally occurring hIAPP functions in synergy with insulin to regulate glucose metabolism [130]. However, the misfolding and deposition of hIAPP generate toxic oligomeric intermediates, which give rise to membrane disruption and ion channel formation in β cells, resulting in β cell dysfunction, insulin resistance, and glucose metabolic disorders in T2DM, thereby worsening the progression of T2DM [131]. Accordingly, inhibitors that counteract the misfolding and aggregation of hIAPP have emerged as a therapeutic avenue for the treatment of type 2 diabetes.

Soluble monomeric hIAPP forms oligomerization and amyloid fibrils through a nucleation-dependent polymerization process. The canonical aggregation process involves a conformational shift of hIAPP from its native, primarily disordered state to an α-helical structure, subsequently adopting a predominant β-sheet fold, culminating with the precipitation of insoluble amyloid fibers [130]. Flavonoids, endowed with significant conformational advantages and spatial hindrance, effectively suppress hIAPP fibrillation, thus impeding the aggregation of hIAPP into oligomers and fibrils enriched in β-pleated sheets [131].

It was validated that IAPP aggregation is triggered by residues 20–29 (SNNFGAILSS), and flavonoids containing aromatic rings could interact with Phe23 of IAPP via π–π stacking, which prevented the binding between Phe23 and itself, thus reducing the β-strand content of IAPP (20–29), resulting in the inhibition of oligomer formation. Moreover, the carbonyl oxygen, hydroxyl, and vicinal hydroxyl group of flavonoids were proven to be beneficial to the suppression of IAPP aggregation [131]. Likewise, amentoflavone and bilobetin exhibited more efficacious inhibitory activity against the misfolding and aggregation of hIAPP than did some monoflavones (luteolin, apigenin), as they displayed a good steric configuration due to their globally symmetrical aromatic structures and hydroxyl groups. They were proven to suppress the self-assembly of hIAPP, depolymerize the aged aggregates into small oligomers and monomers, as well as reverse aberrant folding and mitigate hIAPP-induced cytotoxicity, insulin resistance, and aberrant insulin secretion. Further investigation revealed that the biflavones interfered with the microenvironment around Tyr37, as well as bound with the hydrophobic residues in hIAPP (22–28), especially Phe23, through hydrophobic interaction, hydrogen bonding, and π–π interaction [83]. Baicalein and baicalin maintain the secondary structure of hIAPP in an unfolded α-helix state and β-sheet conformation, respectively [130].

#### 4.1.6. Other Targets

peroxisome proliferator-activated receptor γ (PPARγ): PPARγ can promote insulin sensitivity as a member of the nuclear hormone receptor superfamily [34]. Nigragenon O was found to interact with PPARγ, leading to a decrease in the blood glucose level and improvement in insulin sensitivity in IR 3T3-L1 cells [77]. Astragalin, quercitrin, and quercetin could interact with PPARγ active sites via polar bonding and hydrophobic interactions, as well as elevate liver PPARγ in HFD/STZ-induced diabetic rats [49]. Formononetin could interact with PPARγ through the π–alkyl bond and van der Waals interactions [34], while sulfuretin formed π–π stacking and hydrogen bond interactions [132], thereby boosting insulin sensitivity.

Inflammatory cytokines: T2DM is often accompanied by low inflammation, and there is a vicious cycle between inflammation and insulin resistance, which means insulin resistance-induced hyperglycemia can upregulate inflammation markers and ROS; likewise, increased oxidative stress and chronic low-grade inflammation give rise to insulin resistance and impaired insulin secretion. Consequently, the interventions against inflammation offer a therapeutic avenue for insulin resistance. Additionally, flavonoids, such as myricitrin, astragalin, quercitrin, quercetin [49], and puerarin [39], present anti-inflammation properties via suppressing some inflammatory cytokines involved in the inflammatory response through the nuclear factor kappa-light-chain-enhancer of activated B cells (NF-κB) pathways, such as TNF-α, IL-6, and IL-1β, whose release can impede insulin signaling, causing impaired glucose metabolism and insulin resistance [133].

Adipocytokines: Adipocytokines, containing leptin and adiponectin, are secreted by adipocytes and are involved in maintaining glucose homeostasis owing to the regulation of food and energy consumption, glucose uptake, and insulin sensitivity [134]. The total flavonoids from *Mela stoma dodecandrum* Lour. were found to upregulate adiponectin levels, while downregulating leptin to improve insulin resistance in high-fat-diet-induced rat models [135]. Nigragenon O was found to promote adiponectin secretion in insulin-resistant 3T3-L1 cells, resulting in decreased blood glucose levels and improved insulin resistance [77]. Vitexin and luteolin were considered to interact with adiponectin and its receptor [136].

### 4.2. Signal Pathways

#### 4.2.1. IRS/PI3K/AKT

The insulin-mediated IRS/PI3K/AKT biochemical pathway plays a pivotal role in glucose metabolism through downregulating gluconeogenesis and heightening glycogen synthesis, along with improving glucose utilization.

The binding of insulin to its receptors results in the autophosphorylation and activation of the receptors, which recruits tyrosine to phosphorylate IRS-1/2, leading to tyrosine phosphorylation and the activation of PI3K. This catalyzes the phosphorylation of the 3-hydroxy group of phosphatidylinositol 4,5-bisphosphate (PIP2) to generate phosphatidylinositol 3,4,5-trisphosphate (PIP3). PIP3 then acts as a second messenger, which simultaneously recruits 3-phosphoinositide-dependent protein kinase-1 and -2 (PDK1 and PDX2) and AKT to the plasma membrane, where PDK1 and PDX2 phosphorylate threonine at position 308 and serine at position 473 of AKT, respectively. However, the phosphorylation of IRS at serine prevents IRS from binding to the insulin receptors, resulting in the suppression of the PI3K/Akt pathway [137].

**Figure 3:**
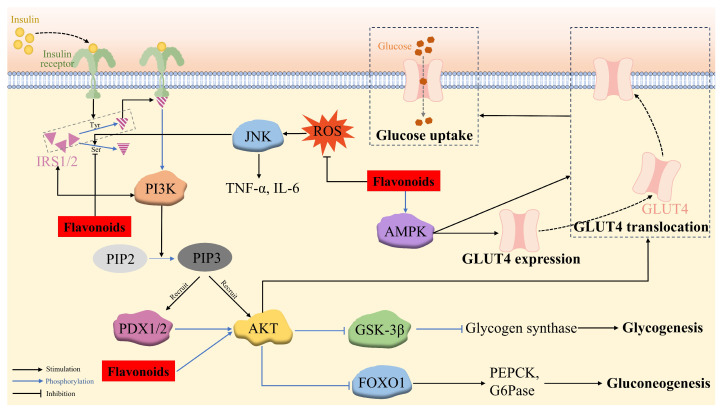
The mechanism of flavonoid regulation of insulin-mediated glucose metabolism via the IRS/PI3K/AKT signaling pathway and insulin-independent glucose uptake via the AMPK pathway, together with ROS/c-Jun N-terminal kinase (JNK) pathway.

The phosphorylated AKT triggers downstream signaling proteins, including forkhead box transcription factor O1 (FOXO1), glycogen synthase kinase-3β (GSK-3β), and GLUT4. FOXO1 is a key transcription factor in regulating glucose production through insulin signaling in the liver, which is localized to the nucleus and enhances hepatic gluconeogenesis by binding to the promoters of the gluconeogenic enzymes (PEPCK and G6Pase). However, P-AKT phosphorylates FOXO1, leading to its dissociation from the nucleus and its decomposition and thereby, the suppression of gluconeogenesis. GSK-3β, a serine protein kinase with an inhibitory effect on glycogen synthesis via phosphorylating glycogen synthase, is deactivated by p-Akt-induced phosphorylation, which inhibits the phosphorylation of glycogen synthase and enhances glycogen synthesis in the hepatocytes [138]. P-AKT also stimulates the translocation of insulin-responsive GLUT4 to the cell surface, thus potentiating glucose uptake in muscle and adipose cells and maintaining blood glucose homeostasis [129,139] (Figure 3).

Several flavonoids have been validated to upregulate the IRS/PI3K/AKT pathway, consequently exerting anti-hyperglycemia and anti-insulin-resistance activity. Quercetin-3-O-α-L-arabinopyranosyl-(1→2)-β-D-glucopyranoside (QAG) stimulated the IRS-1/PI3K/Akt/GSK-3β pathway via suppressing the phosphorylation of IRS-1 at Ser612, resulting in the activation of insulin signaling. Furthermore, the molecular docking result revealed that QAG achieved the above activities by combining with the residues in the active pocket of the insulin receptor [140]. HM-chromanone was able to activate the IRS/PI3K/AKT channel to reduce blood glucose levels and alleviate insulin resistance contributed by the decreased phosphorylation of IRS-1ser307 and elevated GLUT4, p-FOXO1, and p-GSK-3β levels in PA-treated IR HepG2 cells and ob/ob mice [99,100,101]. Naringenin efficiently restored the decreased phosphorylation of Akt at the Ser473 residue site [76]. Astragalin, quercetin-3-arabinoside, and quercetin derived from *Hypericum attenuatum* exhibited appreciable hypoglycemic activity manifested by declined glucose production and accelerated glycogen synthesis in IR HepG-2 cells owing to the decreased expression of PEPCK and G6Pase and the increased expression of glycogen synthase, respectively, via the PI3K/AKT/FOXO1 and PI3K/AKT/GSK3β pathways [139].

#### 4.2.2. AMPK/GLUT4

Skeletal muscle is the principal target organ for insulin-mediated glucose uptake, which is achieved through GLUT4 on its cell membrane. In addition to the insulin-dependent IRS/PI3K/AKT pathway, the translocation of GLUT4 can also be enhanced through an insulin-independent mechanism, namely the AMPK pathway. During this signaling, the AMP-activated protein kinase (AMPK) phosphorylates and deactivates the Akt substrate, detectable at a molecular weight of 160 (AS160), which is a functional Rab-GTPase-activating protein (rab GAP) [141], thus promoting the transcription of the GLUT4 gene and the transposition of GLUT4 to the cell surface, facilitating glucose uptake [61,142]. Therefore, the activation of AMPK/GLUT4 is an effective way to improve insulin sensitivity in T2DM [37]. Licochalcone A alleviated abnormal liver glucose metabolism, partly owing to the activation of AMPK [87]. Formononetin [37] and isorhamnetin [61] were capable of upregulating p-AMPK-α levels, potentiating the expression and translocation of GLUT4. Kaempferol [52] and phlorizin [90] promoted glucose uptake in lipotoxicity-impaired human skeletal muscle cells and mitigated insulin resistance in FFA-treated IR HepG2 cells, respectively, through upregulating p-AMPK and PI3K/Akt/GLUT4 (Figure 3).

#### 4.2.3. ROS/JNK

Insulin resistance is caused by a variety of factors, including ROS-induced oxidative stress. Long-term exposure to FFA, such as palmitic acid, is one of the factors causing mitochondrial dysfunction that leads to excess ROS formation. Increased ROS levels cause oxidative stress and promote the activation of JNK, a stress kinase belonging to the mitogen-activated protein kinase family, which has been reported to be involved in the mechanism of FFA-induced hepatic insulin resistance. The activated JNK pathway facilitates the production of the inflammatory response factors TNF-α and IL-6, as well as IRS-1 phosphorylation at S307, thus inhibiting the PI3K/AKT pathway and providing a mechanism for insulin resistance. HM-chromanone was proven to inhibit ROS production and ROS-induced JNK activation, leading to a significant dose-dependent reduction in TNF-α and IL-6 levels, along with the decreased phosphorylation of IRS-1 at Ser307 and increased AKT/FOXO1 and AKT/GSK-3β levels compared to those of the insulin-resistant HepG2 cells [101] (Figure 3).

#### 4.2.4. PERK/eIF2α/ATF4/CHOP

The endoplasmic reticulum, the vital organelle responsible for the synthesis, proper folding, and secretion of insulin, is highly sensitive to various stress factors that induce misfolded and unfolded proteins to accumulate there, triggering the ER stress and activating the unfolded protein response (UPR) response. However, when the adaptive responses evoked by UPR are inadequate to resume normal cellular function, the apoptosis and autophagy signaling pathways are then triggered. PERK-eIF2α-ATF4-CHOP is an important signaling pathway involved in endoplasmic reticulum stress-induced apoptosis. PERK, an endoplasmic reticulum transmembrane protein acting as a UPR sensor, can phosphorylate eIF2α at ser51, causing the activation of ATF4, followed by CHOP, which gives rise to autophagy and apoptosis during endoplasmic reticulum stress.

Cyanidin-3-O-glucoside remarkably alleviated PA-evoked pancreatic β cell malfunction and apoptosis in a dose-dependent manner through downregulating the ER-stress-related PERK/eIF2α/ATF4/CHOP pathway. It was confirmed that C3G decreased the expression of genes, including *eif2αk3* (PERK) and *ddit3* (CHOP), as well as the expression of PERK, CHOP, glucose regulated protein 78 (GRP78), and GLUT2 and the phosphorylation of PERK and *eif2α* [93].

**Figure 4:**
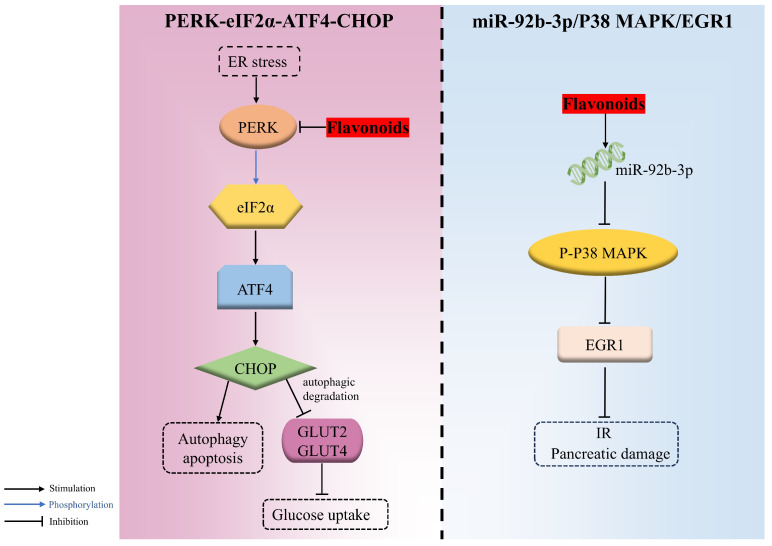
The regulation mechanisms of flavonoids in PERK-eIF2α-ATF4-CHOP and miR-92b-3p/ P38 mitogen-activated protein kinase (P38 MAPK)/ early growth response 1 (EGR1) signaling pathways.

Additionally, the autophagic degradation of two hepatic glucose transporters, namely GLUT2 (insulin-independent) and GLUT4 (insulin-dependent), during hyperglycemia was motivated via PERK/eIF2α/ATF4/CHOP, thereupon decreasing glucose uptake. Nevertheless, morin was proven to ameliorate the hyperglycemia and insulin sensitivity attributed to the repression of PERK-adjusted transporter degradation and the boosted expression of glucose transporter proteins in both HepG2 cells and diabetic rats [143] (Figure 4).

#### 4.2.5. miR-92b-3p/P38 MAPK/EGR1

miR-92b-3p is the dominant form during miRNA formation, which inhibits the phosphorylation of P38 MAPK and then suppresses EGR1 expression, which is associated with β-cell proliferation and transactivation of the insulin gene. EGR1, a stress-responsive gene highly expressed during diabetes, is responsible for the release of inflammatory factors that can cause IR. It was demonstrated that quercetin was capable of upregulating miR-92b-3p, thus decreasing the activation of P38 MAPK and further repressing EGR1 expression, which resulted in decreased blood glucose and the mitigation of IR and pancreatic damage in HFD/STZ-induced T2DM mice [45] (Figure 4).

### 4.3. Gut Microbiota

The human gut contains trillions of microorganisms, encompassing over 1000 species of bacteria [144]. The intestinal flora is implicated in the release of intestinal hormones, bile acid metabolism, insulin sensitivity regulation, and lipid metabolism disorder, and its metabolites are linked to the functionality of remote organs and systems via the circulatory system. In healthy individuals, the gut microbiota maintains a dynamic equilibrium. Abnormalities in the abundance and proportions of bacteria can lead to an imbalance of the gut microbiota, resulting in intestinal disorders, as well as various metabolic disorders such as diabetes, obesity, cardiovascular diseases, and non-alcoholic fatty liver disease. Numerous studies indicate that diabetes is accompanied by alterations in gut microbiota composition [51]. Accordingly, restoring disrupted gut microbiota and promoting the growth of hypoglycemic probiotics are effective strategies for treating diabetes.

**Figure 5:**
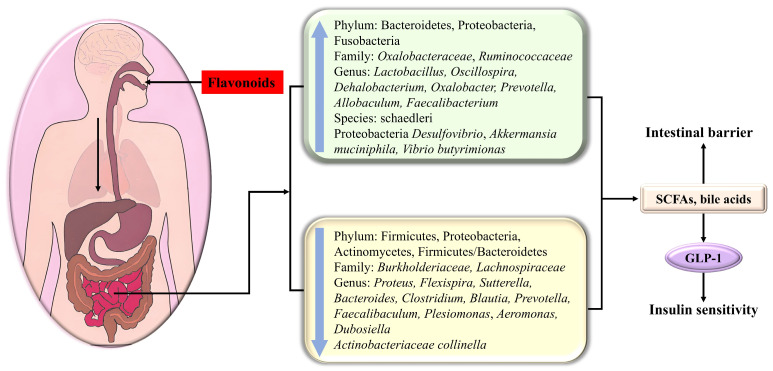
The flavonoid modulation of glucose metabolism via adjusting the composition of intestinal flora and its metabolites.

A significant number of studies have declared that the low bioavailability of flavonoids affords ample time for interaction with the intestinal tract, which effectively modulates the composition of gut microbiota and its metabolites and protects the intestinal barrier [145] (Figure 5). Therefore, flavonoids represent potentially efficacious agents for regulating disruptions in the gut microbiota.

Diabetes usually leads to the rarefication of the dominant flora Bacteroidetes, which participate in some vital metabolism roles, including carbohydrate fermentation, i.e., the increment of Firmicutes, Proteobacteria (containing various pathogens), Actinomycetes, and Firmicutes/Bacteroidetes. Watanabe et al. indicated that the addition of soy isoflavone could decrease the ratio of Firmicutes/Bacteroidetes [146], which was beneficial for glycemic control and GLP-1 secretion.

Kaempferol had the potential to significantly decrease fasting blood glucose and ameliorate glucose tolerance and insulin sensitivity as a consequence of reversing the abnormal alterations of the gut microbiota in HFD-treated mice compared with that of the control group, including the reduction in the Firmicute *Dehalobacterium*, the Proteobacteria Desulfovibrio, as well as in *Proteus flexispira* and *Sutterella*; the increment of *Actinobacteriaceae collinella*, *Oscillospira*, *Dehalobacterium*, and *schaedleri* species; and the increase in the Firmicute *Vibrio butyricimonas*. In the microbiotas studied, *Collinsella* is related to insulin, and the increase in *Desulfovibrio* can improve glucose metabolism [51]. Nobiletin enhanced the abundance of *Allobaculum*, which was indicated to be an active glucose assimilator and could improve energy homeostasis in HFD-fed mice [25]. Neohesperidin dihydrochalcone had the potential to bolster glycogenesis and restrain gluconeogenesis in the liver of diabetic zebrafish as a consequence of upregulating *Prevotella* [147].

Apigenin increases *Akkermansia* and decreases *Faecalibaculum* and *Dubosiella* at the genus level [148]. Cyanidin-3-O-glucoside (C3G) demonstrated efficacy in treating hyperglycemia through adjusting the intestinal flora distribution, manifested by the decline in Firmicutes/Bacteroidetes, *Burkholderiaceae*, *Lactobacillus*, and Bacteroides and the increase in *Oxalobacteraceae*, *Oxalobacter*, *Prevotellaceae*, and *Helicobacter* [92]. HMF (3,3′,4′,5,6,7,8-heptamethoxyflavone) prominently changed the composition, function, and metabolism of the gut microbiota, especially *Faecalibaculum rodentium*, *Collinsella aerofaciens* and *Lactobacillus fermentum*, in HFD-fed mice, thus displaying a beneficial effect on metabolic syndrome [27]. Isoxanthohumol, a prenylated flavanone found in beer hops, markedly influenced the gut microbiota composition, specifically promoting the growth and abundance of *Akkermansia muciniphila*, while inhibiting some *Bacteroides* and *Clostridiums* [149]. Quercetin decreased the abundance of Proteobacteria, Bacteroides, Escherichia-Shigella, and Escherichia coli in db/db mice, thereby reducing glucose and alleviating insulin resistance [150].

In addition to causing compositional changes, metabolites produced by the gut microbiota also have a striking impact on T2DM. For instance, short-chain fatty acids (SCFAs) derived from polysaccharide fermentation heighten insulin sensitivity and deplete blood sugar levels by modulating the secretion of the gastrointestinal hormone GLP-1. Additionally, bile acids, involved in food digestion, serve as crucial signaling molecules impacting various metabolic disorders, including cancer, obesity, and type 2 diabetes. Secondary bile acids can engage with the G protein-coupled receptor 5 on intestinal epithelial L cells, thereby stimulating the release of GLP-1 to modulate glucose metabolism [92]. Dietary flavonoids can modulate microbial metabolites, subsequently impacting host metabolism.

Neohesperidin dihydrochalcone significantly alleviated insulin resistance due to the promotion of SCFAs and the suppression of pro-inflammatory cytokines contributed by the decrease in harmful bacteria, including Proteobacteria, *Plesiomonas*, and *Aeromonas*, together with the increase in *Faecalibacterium* and Fusobacteria. Among the aforementioned bacteria, *Faecalibacterium* has the potential to ferment glucose to produce SCFAs, including butyric acid, formic acid, and D-lactic acid, meanwhile boosting glycogenesis [147]. Kaempferol upregulated *Vibrio butyrimionas*, which can generate short-chain fatty acids targeting the intestines, liver, and other organs to improve intestinal health, blood glucose, and insulin resistance [51]. The addition of soy isoflavone could enhance the abundance of short-chain fatty acid-producing bacteria in intestinal bacteria [146]. HMF (3,3′,4′,5,6,7,8-heptamethoxyflavone) enhanced short-chain fatty acid- and bile acid-producing bacteria and inhibited harmful bacteria, thereby promoting bile acid metabolism and reducing fatty acid metabolism and inflammatory response [27]. C3G enriched the abundance of the microbiomes involved in SCFA biosynthesis and bile acid metabolism in diabetic db/db mice, giving rise to the expansion of short-chain fatty acids (propanoic acid, isobutyric acid, butyric acid) and bile acids, followed by the secretion of GLP-1, which in turn bolstered insulin release, lowering blood glucose [92].

Noteworthily, isoxanthohumol enhanced the intestinal barrier protective function manifested by increased mucin layer thickness and claudin-1 (a tight-junction-related protein in the colon), which might be contributed by the *A. muciniphila*-induced enhanced turnover of epithelial cells or the production of SCFAs (the main nutrient for intestinal cells) [149]. Neohesperidin dihydrochalcone adjusted the composition of intestinal flora and thus improved the functionality of the intestinal barrier in diabetic zebrafish [147].

## 5. Synergistic Hypoglycemic Effects of Flavonoids

Quercetin could also be useful in combination with other drugs to potentially enhance the effects or synergistically interact with them in order to reduce their side effects and related toxicity.

Several studies have suggested that the combination of different flavonoids can synergistically alleviate diabetes, showing surprising effects. It was demonstrated that the addition of curcumin and baicalein (75 mg/kg each) remarkedly diminished FBG (13.30 ± 2.97 mmol/L) in mice fed with a high-fat diet for 4 weeks compared with the results for model diabetic group (25.49 ± 9.28 mmol/L). Additionally, the ability of the combination to decrease FBG was superior to that of individual 150 mg/kg curcumin (21.91 ± 9.08 mmol/L) or 150 mg/kg baicalein (25.85 ± 9.41 mmol/L) treatments. The common gene targets and the kyoto encyclopedia of genes and genomes (KEGG) pathway might be decisive in elucidating the mechanisms of the synergistic effect of curcumin and baicalein [151].

Koh et al. revealed that the combination of myricetin (40 μM) and luteolin (100 μM) required to produce a 50% inhibition of the activity of α-glucosidase was 6.41 ± 0.17 μM myricetin and 16.02 ± 0.43 μM luteolin, suggesting a synergistic inhibition [152]. The combinations of apigenin (5, 10, 15 μM) and nepetin/scutellarein/hispidulin (5, 10, 15 μM) were able to synergistically repress the activity of α-amylase apart from the conjunction of apigenin (5 μM) and hispidulin (5 μM), which exerted additional effect. Since apigenin was a non-competitive inhibitor of α-amylase, while the other three flavonoids were competitive inhibitors, they could interact with different sites of α-amylase, displaying a synergistical inhibitory effect [111].

Scarpa et al. elucidated that naringenin and hesperetin were able to synergistically increase insulin receptor (INSR) mRNA expression in insulin-resistant HepG2 and Hep3B cells, synergistically reduce GLUT3 mRNA levels in IR Hep3B cells, and synergistically elevate sirtuin 1 (SIRT1) expression in IR HepG2 cells. Furthermore, naringenin, hesperetin, curcumin, polydatin, and quercetin presented a synergistic inhibition against SEMA3E mRNA expression in insulin-evoked IR HepG2 cells and DPP-IV catalytic activity in IR Hep3B cells, suggesting an effective novel strategy for the treatment of T2DM [153]. The combined treatment with 25 mg/kg kaempferol and 25 mg/kg myricetin decreased body weight, glycogen level, α-amylase activity, pro-inflammatory cytokines (IL-6, IL-1β, and TNF-α), and elevated insulin level more significantly than the addition with 50 mg/kg kaempferol or 50 mg/kg myricetin alone in STZ-induced diabetic rats, suggesting that co-treatment with kaempferol and myricetin exerted a better hypoglycemic effect than did treatment with one of the compounds independently [154].

## 6. Limitations in the Application of Flavonoids

### 6.1. Absorption and Bioavailability of Flavonoids

Despite the objective hypoglycemic activities, flavonoids generally exhibit low oral bioavailability due to their poor water solubility. The bioavailability of quercetin is often relatively low (<10%) and is influenced by the type of sugar moiety [155], by which its hypoglycemic activity is also affected. In addition, because of massive free hydroxyl groups, flavonoids are easily glucuronidated and sulfated in the intestinal cells, and then expelled by efflux transporters, resulting in poor and unstable oral absorption. Moreover, flavonoids are also sensitive to various environmental stresses (heat, light, pH) due to the presence of hydroxyl, ketone, and unsaturated double bonds. This may lead to degradation or biotransformation during storage and systemic circulation, severely limiting their efficacy through oral absorption [156].

In conclusion, the clinical application of flavonoids is greatly limited. To address these issues, a number of promising strategies have been developed and applied, such as the use of absorption enhancers (e.g., apple pectin, chitosan), structural transformations (e.g., prodrugs, glycosylation), the glycosylation of flavonoids (e.g., chemical synthesis, enzyme pathway manipulation), and pharmaceutical techniques (e.g., carrier complexes, nanotechnology, cocrystals) [157] to deliver poorly water-soluble flavonoid. Using these strategies alone or in combination effectively improves the oral bioavailability of flavonoids, prevents its degradation or metabolism in the gastrointestinal tract, or delivers it directly to the targets. In the future, we should pay more attention to the toxicity and safety of these modified oral preparations, accelerate the translation of results and clinical research, and promote the clinical application of flavonoids for lowering blood glucose.

### 6.2. Toxicity and Safety of Flavonoids

Although flavonoids have shown significant potential for improving glucose metabolism, recent research indicates a growing concern about the endocrine disruption, developmental toxicity, and mutagenicity of many flavonoids.

Anthocyanins are widely found in plants, fruits, and vegetables and are generally safe to consume via a normal dietary intake. According to early toxicological studies, anthocyanin-rich extracts exhibit very low mutagenicity, reproductive toxicity, teratogenicity, and acute and short-term toxicity. However, data on the long-term toxicity and carcinogenic effects of anthocyanins are still lacking [158].

Luteolin, apigenin (flavones), quercetin (flavonol), and genistein (isoflavone) showed specific estrogenic activity, developmental toxicity, and mutagenicity. Luteolin displayed higher developmental toxicity and genotoxicity than did apigenin, and quercetin exhibited lower developmental toxicity and estrogenic activity among the flavonoids. Luteolin and genistein showed high developmental toxicity (45–477 μg/kg), with up to 50% mortality in the chicken embryo test [159]. A toxicity study of two isoflavones, genistein and daidzein, showed that the hatching rate of zebrafish larvae was significantly reduced at 72–96 h post fertilization (hpf) at the doses of 20 mg of daidzein and 10 mg of genistein. Genistein showed higher toxicity than did daidzein, often inducing greater transcriptional responses, as the LC50 of genistein estimated at 96 hpf was 4.4 mg/L, which is significantly lower than that of daidzein (over 65.15 mg/L) [160].

Supplementation with 800 mg of EGCG daily for 5 days did not cause liver injury or abnormal serum folate levels in 39 women aged 18 to 40 years [161]. However, high doses of dietary EGCG supplements may be associated with certain health risks. An acute toxicity study of EGCG showed that EGCG doses exceeding 30 mg/L resulted in 100% mortality and organ-specific toxicity. Continuous exposure to high concentrations of EGCG may cause severe tissue changes in experimental animals [162]. EGCG showed a weak degradation effect on 5-methenyltetrahydrofolic acid (5-MTHF), the active form of folate, which can be used directly by the body. In serum, 2 μm EGCG had no significant effect on 5-MTHF within 48 h, but 0.1 mM and 1 mM EGCG showed a pro-degradation effect on 5-MTHF from 6 h, and 20 μm EGCG decreased the concentration of 5-MTHF for 48 h [163].

Quercetin treatment for 24 h did not affect the cellular metabolic activity of MCF7 cells but showed cytotoxic effects after 48 h and 72 h of treatment, with IC_50_ values of 43.18 μg/mL and 18.49 μg/mL, respectively. The results of live cell imaging showed that no apoptosis was found after 24 h, but prolonged exposure reduced the apoptosis-promoting effect of quercetin [164].

A toxicological study on biflavonoids from *Ginkgo biloba* leaves (amentoflavone, sciadopitysin, ginkgetin, isoginkgetin, and bilobetin) showed that all five flavonoids reduced cell survival of human renal tubular epithelial cells and human normal hepatocytes in a dose-dependent manner, suggesting potential hepatorenal toxicity. After intragastrical administration at a dose of 20 mg/kg per day for 7 days, the activity of alkaline phosphatase was significantly increased, and all five biflavonoids induced acute kidney injury in treated mice. More attention should be paid to ensure the safety of *Ginkgo biloba* preparations [165].

Given the above facts, the supplementation of flavonoids should be scientific and cautious to maximize their health benefits and avoid potential risks. A large number of studies are still needed in the future to fully elucidate the potential toxicity of flavonoids, especially their safety under long-term or low-dose conditions, which still requires careful evaluation. The relationship between toxicity and the structure of flavonoids should also be additionally studied to guide its further modification and application.

## 7. Conclusions and Future Perspectives

As the incidence of diabetes continues to rise around the world, there is increasing interest in natural plant-derived flavonoids exhibiting low side effects and considerable anti-diabetes activity. The subclasses of hypoglycemic flavonoids discussed in this review comprise flavones, isoflavones, flavonols, flavanols, flavanones, flavanonols, biflavonoids, chalcones, anthocyanins, and homoisoflavonoids, which exhibit therapeutic properties against diabetes through a variety of targets and pathways, e.g., α-amylase, α-glucosidase, gluconeogenic enzymes, DPP-IV, estrogen receptors, GLUT, IRS/PI3K/AKT, AMPK/GLUT4, PERK/eIF2α/ATF4, etc. In addition, flavonoids also modulate abnormal gut microbiota to alleviate the symptoms of diabetes. Moreover, with further research regarding the hypoglycemic mechanism of flavonoids, the synergistic effects between flavonoids have also received widespread attention; however, the exploration of this aspect is relatively fractional and lacks further investigation and validation regarding the mechanism of the synergistic action between related targets. Overall, flavonoids offer a new insight into hypoglycemic therapy.

Of particular note, a substantial number of studies have focused only on the hypoglycemic activity of total flavonoids derived from the plant extract, without further identifying and validating the exact physiological activity of the flavonoid contained therein, which is not conducive to the symptomatic use and clinical application of flavonoids. Moreover, numerous studies on the hypoglycemic effects of flavonoids remain confined to the in silico phase, without subsequent in vivo validation. Previous studies have detected that flavonoids displaying carbohydrate metabolism enzyme inhibitory activity in silico were unable to significantly suppress carbohydrate absorption in vivo [117]. Therefore, complementing the in vitro characterization with in vivo experiments is certainly necessary. For in vivo experiments, while many animal models of diabetes exist, more convincing human experimental data are still lacking. However, most studies are conducted in vitro, and further in vivo and clinical studies should determine the efficacy and safety of flavonoids. With the development of bioavailability research, increasing attention has been paid to the physiological function of flavonoid metabolites. Flavonoids may exhibit different positive effects at different stages. In the future, we should further study the cellular sites and various signaling pathways that its decomposition products may reach, as well as the effects on the structure and function of gut microbiota.

## Figures and Tables

**Table 1:** The molecular interactions of flavonoids with α-amylase.

Flavonoids	IC_50_	Interacted Amino Acid Residues	Intermolecular Interactions
apigenin [111]	21.66 ± 0.96 μM	Trp396, Lys457, Ala489, His491, Glu493	hydrogen bonds, hydrophobic interactions, π-interactions
scutellarein [111]	14.99 ± 0.62 μM	Trp151, Lys200, His201, Glu233, Ile235, Glu240	hydrogen bonds, π-interactions
nepetin [111]	10.83 ± 0.49 μM	Gln63, Asp197	hydrogen bonds, π-interactions
hispidulin [111]	30.08 ± 1.12 μM	Trp59, Tyr62, Gln63, Asp197, Asp300	hydrogen bonds, π-interactions
delphinidin 3,5-O-diglucoside [113]	/	Trp59, Gln63, Asp300, His305, Asp356	hydrogen bonds, π-cation interaction
malvin [113]	/	Trp59, Gln63, Tyr151, Glu233, Asp300, His305	hydrogen bonds, π-cation interaction
nasunin [113]	/	Trp59, Gln63, Asp147, Asp300, His305	hydrogen bonds, π-cation interaction
cyanidin-3-(6-acetylglucoside) [113]	/	Trp59 Gln63, Glu233, His305, Asp300, Asp 336	hydrogen bonds, π-cation interaction
cyanidin 3-O-xylosyl-rutinoside [113]	/	Trp59, Gln63, Thr163, Glu233, His305, Asp356	hydrogen bonds, π-cation interaction
EGCG [65]	0.548 ± 0.029 mg/mL	Arg195, Ala198, His201, Glu233, His299, Tyr62, Leu165, His10	hydrogen bonds, hydrophobic interactions, salt bridges
swertisin [32]	/	Phe280, Phe321, His325	hydrogen bonds, carbon–hydrogen bonds

**Table 2:** The molecular interactions of flavonoids with α-glucosidase.

Flavonoids	IC_50_	Interacted Amino Acid Residues	Intermolecular Interactions
luteolin [21]	32.3 μM	N283, T287, Y286	van der Waals forces, hydrogen bonds, π−π stacking
scutellarein [118]	2.4 ± 0.2 μM	Lys156, Ser241, Ser311, Asp307, Arg315, Lys213, His280	hydrogen bonds, π-interactions
nepetin [118]	11.8 ±0.2 μM	Lys156, Gly161, Asp233, Asn235, Phe314, Asn317, Ala418, Glu422, His423	hydrogen bonds, π-interactions
apigenin [118]	14.3 ± 0.2 μM	Gly-161, Asp-233, Asn-235, Ala-418, Ile-419, Glu-422, His-423	hydrogen bonds, hydrophobic interactions, π-interactions
hispidulin [118]	32.1 ± 0.3 μM	Lys156, Gly161, Phe314, Asn317, Ala418, Ile419, Glu422, His423, Glu429	hydrogen bonds, hydrophobic interactions, π-interactions
strychnobiflavone [85]	/	Arg315, Phe303	cation-π and π–π stacking,
hesperidin [72]	18.52 μM	Arg422, Asn424, Arg467, Trp709,	five hydrogen bonds, hydrophobic interactions
apigenin [119]	0.525 mM	THR141, RG430, PRO466, ARG513, ILE517, ALA518	hydrogen bonds, electrostatic interaction, hydrophobic interactions
cyanidin-3-O-glucoside [94]	/	Leu313, Ser157, Tyr158, Phe314, Arg315, Asp307	hydrogen bonds, hydrophobic interaction
lutexin [24]	0.13 mg/mL	Asp69, Arg315, His351, Asp215, Glu277, Phe303, Asp352, Glu411, Arg442, Arg446	carbon–hydrogen bond, hydrogen bonds
swertisin [32]	/	Tyr63, Asp98, Asn258, Phe280, Glu323; Arg197, Asp326; Phe321, His325	hydrogen bonds, π–cation interactions, carbon–hydrogen bonds, van der Waals interactions

## Supplementary Materials

The following supporting information can be downloaded at: https://www.mdpi.com/article/10.3390/antiox14040378/s1, Table S1: The subclasses of flavonoids and their core carbon skeleton; representative flavonoids; Table S2: The hypoglycemic effects of flavonoids studied in recent years. Refs. [166,167,168,169,170] are cited in the Supplementary Table S2.

## Abbreviations

The following abbreviations are used in this manuscript:

DM	diabetes mellitus
T1DM	type 1 diabetes
T2DM	type 2 diabetes
IR	insulin resistance
FBG	fasting blood glucose
STZ	streptozocin
HFD	high-fat diet
PA	palmitic acid
GSIS	glucose-stimulated insulin secretion
EGCG	epigallocatechin gallate
ROS	reactive oxygen species
FINS	fasting insulin
C3G	cyanidin-3-O-glucoside
HbA1c	glycosylated hemoglobin
AGE	advanced glycation end-product
G6Pase	glucose-6-phosphatase
PEPCK	phosphoenolpyruvate carboxykinase
GCK	glucokinase
GKRP	glucokinase regulatory protein
DYRK1A	dual specificity tyrosine phosphorylation-regulated kinase 1 A
DPP-IV	dipeptidyl peptidase IV
Drak2	death-associated protein kinase-related apoptosis-inducing kinase-2
mTOR	mammalian target of rapamycin
PTP1B	protein tyrosine phosphatase 1B
GIP	glucose-dependent insulinotropic polypeptide
GLP-1	glucagon-like peptide-1
ER	estrogen receptor
SGLT	sodium-dependent glucose transporter
GLUT	facilitative glucose transporter
hIAPP	human islet amyloid polypeptide
TNF-α	tumor necrosis factor-α
IL-6	interleukin 6
FFA	free fatty acids
SCFAs	short-chain fatty acids
