# Supplementary material for: Research Progress on Hypoglycemic Effects and Molecular Mechanisms of Flavonoids: A Review
Source: Antioxidants (Basel). 2025 Mar 22;14(4):378. doi: 10.3390/antiox14040378 (PMC12024137; doi:10.3390/antiox14040378)
Supplement: Supplementary file 1 [file antioxidants-14-00378-s001.zip › antioxidants-3468438-supplementary.pdf]

Table S1. The subclasses of flavonoids and their core carbon skeleton, representative flavonoids.

Subclass	Core Skeleton	Flavonoids
Flavones	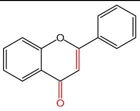	acacetin, apigenin, nobiletin, baicalein, luteolin
Isoflavones	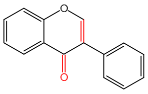	biochanin A, daidzein, formononetin, puerarin, genistein
Flavonols	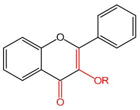	quercetin, rutin, silibinin kaempferol, myricetin fisetin, morin, epimedin C
Flavanols	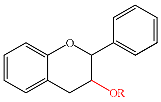	EGCG, (-)-Epicatechin-6, Catechin
Flavanones	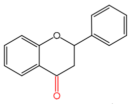	hesperetin, naringenin, eriodictyol, isosakuranetin，
Flavanonols	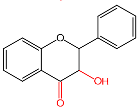	dihydromyricetin, taxifolin, sanggenon C
Chalcones	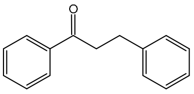	licochalcone A, neohesperidin, nothofagin, phlorizin
Anthocyanins	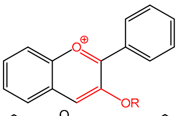	cyanidin, cyanidin-3-O-glucoside, delphinidin, malvidin-3-arabinoside
Homoisoflavonoids	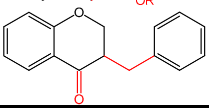	HM-chromanone

Table S2. The hypoglycemic effects of flavonoids studied in recent years.

Flavonoids		Structure	Dosage	Mechanism and relevant indicators	Model			Sources
					In vitro	*In vivo*	*In silico*	
	Acacetin	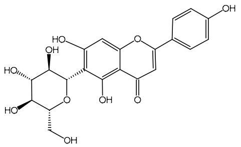	10, 15, 20, 25μM;<br>25, 50, 100 μM;	↓ROS, blood glucose, liver damage, pancreas damage, FBG, lipid, inflammation (TNFα, IL-6, IL-8, MDA)<br>↑antioxidant enzymes (CAT, SOD, GPx), body weight	RINm5F cells	Zebrafish;<br>rat		*Saussurea involucrate, Ziziphora clinopodioides Lam, Robinia pseudoacacia*<br>[12-13]
Flavones	Apigenin (4′,5,7-trihydroxy flavone)	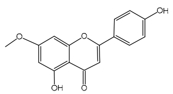	6.25, 12.5 μmol/L;<br>10 μM	↓inflammation, IR, hepatic TG and TC, IL-1β, TNF-α, ROS, AGEs, p-P65, α-glucosidase, α-amylase<br>*Faecalibaculum, Dubosiella*<br>↑glucose tolerance, glycogenesis, glucose consumption, GLUT4, IRS-Akt-GLUT4/GSK-3β<br>*Akkermansia*	HepG2 cells	C57BL/6 J mice	Molecular docking, Molecular dynamic simulation	Celery, parsley, grapefruit, onions, orange, tea, chamomile, and others [32,119,148]
	Vitexin (apigenin-8-C-glucoside)	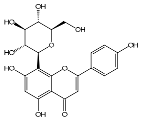	/	↓α-glucosidase, α-amylase, post-prandial plasma glucose, ROS, inflammation, IR<br>↑adiponectin, IRS/PI3K/AKT	HepG2 cells		molecular docking	*Feronia elephantum*<br>[132,136,152,166]
	Isovitexin (apigenin-6-C-glucoside)	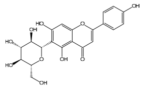	5 mg/kg	↓AGEs, FBG, ROS, inflammation, IR<br>↑IRS/PI3K/AKT	HepG2 cells	albino Wistar rats		*Aspalathus linearis* (Burman f.) R. Dahlgren, *Pterocarpus Santalinoides* [89,166-167]
Flavones	Baicalein	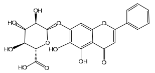	5, 10 μM<br>12.5, 25 μmol/L	↓hIAPP fibril, glycation (AGEs, BSA glycation, glycated albumin), free radicals, α-glucosidase, ROS, p-P65<br>↑glucose consumption, glycogenesis, IRS-Akt-GLUT4/GSK-3β	INS-1 rat cells;<br>HepG2 cells			*Scutellaria baicalensis* Georgi<br>[15,130,138]
	Baicalin	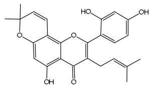	5, 10 μM	↓hIAPP fibril, glycation (AGEs, BSA glycation, glycated albumin), free radicals, α-glucosidase, CYP7A1	INS-1 rat cells			*Scutellaria baicalensis* Georgi [14-15,130]

	Luteolin	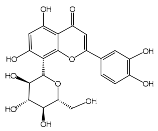	5, 10, 25, 40, 50 μM; 2.5, 5, 10μM; 6.25, 12.5 μmol/L; 5, 20 mg/kg; 100, 400 mg/kg	↓α-amylase, α-glucosidase, cyclooxygenase, ROS, AGEs, p-P65, *Drak2*, β cell apoptosis (PARP, cleaved caspase3, cleaved caspase9), FBG, ALT, TG, blood glucose<br>↑glucose consumption, glycogenesis, IRS-Akt-GLUT4/GSK-3β, GSIS, autophagy, glucose tolerance	HepG2 cells; INS-1E cells	albino Wistar rats; C57 mice		*Thespesia garckeana* F. Hoffm. (Snot Apple), *Rumex vescarius*, *Vernonia amygdalina* [17-22,138]
	Luteolin-7-O-rutinoside	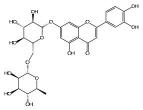	20 μM	↓p-IRS-1/IRS-1, ROS<br>↑insulin secretion, glucose uptake	RIN-5F cells, L6 myotubes			Mentha longifolia L., Olea europaeaL. [23]
	Lutexin	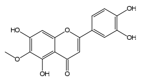	/	↓α-glucosidase			Molecular Docking, Molecular Dynamics	*Itea omeiensis* [24]

Flavones	Nobiletin	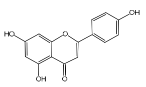	10, 12μM; 100, 150 mg/kg	↓glucose, LPS, islet injuries, inflammation (TLR-4/P-P65/TNF-α, NLRP3/Caspase-1/IL-1β), apoptosis (Bcl-2/BAX/Caspase-3), macrophage accumulation, Firmicutes/Bacteroidetes, *Clostridium_XIVa* ↑insulin, TNF-α, IL-1β, mitophagy (Pink-1/Parkin/LC3), mitochondrial membrane potential *Alloprevotella*, *Parabacteroides*, *Prevotella*, *Desulfovibrio*	NIT-1 cells	C57/BL6 mice	Molecular Docking, Molecular modeling, molecular dynamics simulation	Orange, lemon [25]
	Genkwanin	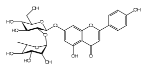	/	↓α-glucosidase		/		Vietnamese *Aquilaria crassna* [29]
	Isosinensetin (3′,4′,5,7,8-pentamethoxy-flavone)	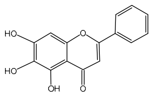	/	↓PTP1B		/		*Citrus*, *Poncirus trifoliata* (L.) Raf. [168]
	Morusin	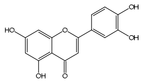	50 mg/kg	↓blood glucose		ICR mice		*Morus alba* L. [28]
Flavones	Eupafolin	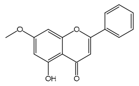	60 mg/kg	↓FBG, insulin, IR, TG, TC, LDL, VLDL, TC/HDL, oxidative stress (TBARS, GST, GSH, catalase, SOD, total thiols), α-glucosidase ↑HDL, InsR, IRS-2, GLUT4, PPAR-γ, PI3K/Akt		Wistar rats		*Artemisia princeps*, *Eremophila denticulate* [16,30]
	Tectochrysin	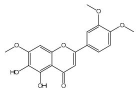	10, 20 mg/kg; 40 μmol/L	↓glucose, gluconeogenesis, lipolysis (adipose triglyceride lipase, hormone-sensitive triglyceride lipase), insulin resistance ↑insulin sensitivity, glucose tolerance, glucose uptake, glycogen, p-IRβ, p-IRS1, p-Akt	3T3-L1 preadipocytes; C2C12 myoblasts	db/db mice; C57BL/6J mice		Propolis, *Alpinia oxyphylla*, *Muntingia calabura*, *Carya genus* [26]
Flavones	Oroxylin A (5,7-dihydroxy-6-methoxy flavone)	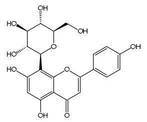	/	↓α-glucosidase, α-amylase			molecular docking	*Oroxylum indicum* (Linn.) Bentham ex Kurz [31]
	3,3',4',5,6,7,8-	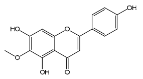	/	↓body-weight gain, fat accumulation, hepatic steatosis, inflammation		Mice (HFD)		[27]

	heptamethoxyflavone			↑short-chain fatty acid (SCFA)- and bile acid-producing beneficial bacteria, SCFAs, bile acids			
	Hispidulin	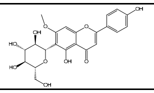	/	↓PTP1B	/		*Eremophila clarkei* [124]
Flavones	Swertisin (7-O-methylapigenin)	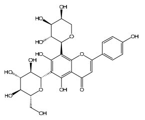	/	↓AGEs, α-glucosidase, α-amylase		Molecular docking, Molecular dynamic simulation	*Carex fraseriana* [32]
Flavones	Schaftoside	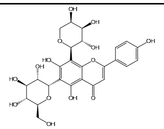	/	↓DPP-IV, α-glucosidase, α-amylase		Molecular docking	Sugarcane [33]
Isoflavones	Biochanin A	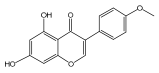	10 mg/kg	↓plasma glucose, IR, glycated hemoglobin, G6Pase, fructose-1,6-bisphosphatase, glycogen phosphorylase, liver damages, pancreatic injuries<br>↑plasma insulin, glucose tolerance, hemoglobin, hexokinase, glycogen synthase	Sprague-Dawley rats		Soy, peanuts, chickpea, alfalfa sprouts [35]
Isoflavones	Daidzein	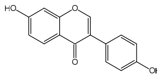	25, 50, 100 mg/kg	↓plasma glucose, bladder weight,<br>↑body weight, antioxidant enzyme (GSH, SOD, CAT)	Sprague Dawley rats		Soy [36]

Isoflavones	Formononetin	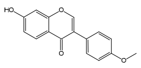	20 mg/kg; 2.5, 5, 10 mg/kg, 3.125, 6.25, 12.5 μM	↓serum glucose, insulin resistance, fasting glucose level, oxidative stress, LDL, TG, islet damage, DNA damage, cell apoptosis, ROS<br>↑serum insulin, AMPK/GLUT4, Keap1/Nrf2, cell proliferation	MIN6 cells	C57BL/6J mice	Molecular docking, Molecular dynamics simulation	*Astragalus membranaceus*, *Trifolium pratense* L. and *Pueraria lobata* (Willd.) [37-38]
Isoflavones	Puerarin	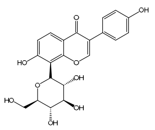	200 mg/kg; 75 μM	↓body weight, hyperlipidemia (TG, TC), oxidative stress (↓ ROS, MDA; ↑ GSH-Px, CAT, SOD), liver dysfunction (ALT, AST), liver inflammation (↓ IL-1β, IL-6, TNF-α, p-p65; ↑ Nrf2 nuclear translocation), liver injury<br>↑glucose tolerance, insulin sensitivity, SIRT1/Nrf2	The α mouse liver 12 (AML12) cell line	C57BL/6 J mice	Molecular docking,	Kudzu root [39]

Flavonols	Quercetin	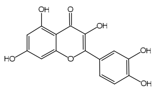	1.0^{-6}, 1.0^{-5}, 1.0^{-4}, 1.0^{-3}, 1.0^{-2} g/kg; 50 mg/kg; 120 mg/kg/d	↓FBG, inflammation (IL-6, TNF-α, IL-1β), blood glucose, PEPCK, G6Pase, IR, TG, TC, LDL, hepatic and pancreatic injuries, PTP1B, α-glucosidase, NF-kB p65, insulin, HOMA-IR, HbA1c, p-P38 MAPK<br>↑body weight, insulin, glucose tolerance, glycogen synthase, antioxidant enzymes (CAT, SOD, GST, GSH), HDL, PI3K-AKT-FOXO1/GSK3β/GLUT4, PPAR-α/γ, p-AMPK, hexokinase, miR-92b-3p/EGR1	HepG2 cells	Wistar albino rats; ICR mice; C57BL/6 mice	Molecular Docking	*Hypericum attenuatum, Abelmoschus esculentus (L.) Moench, Euphorbia peplus* [43-45,47-49,139]
	Rutin (quercetin-3-O-β-rutinoside)	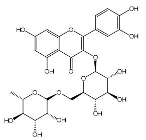	100 μg/mL	↓α-glucosidase, α-amylase, aldose reductase, protein glycation (fructosamines, protein carbonyl compounds, AGEs), free radical (DPPH, ABTS, superoxide)<br>↑glucokinase, antioxidant enzymes, quality of life			Molecular Docking, molecular dynamics simulation	Whole jackfruit flour, *Artemisia princeps* [121,169-170]
Flavonols	Quercitrin (quercetin 3-rhamnoside)	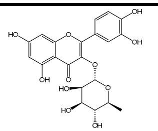	/	↓liver NF-kB p65, TNF-α, IL-1β<br>↑hexokinase, liver PPARγ		Wistar rats	Molecular Docking	*Euphorbia peplus* [49]
Flavonols	QAG	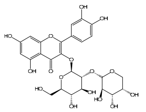	/	↓↑glucose uptake, glycogen synthesis, GLUT2, GLUT4, IRS/PI3K/Akt/GSK-3β	HepG2 cells			*Eucommia ulmoides* [140]
	Quercimeritrin	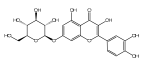	100, 200 mg/kg	↓α-glucosidase, α-amylase, postprandial blood glucose		db/db mice	Molecular Docking, Molecular Dynamics Study	*Astragalus creticus* [115]
	Isorhamnetin	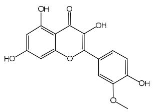	/	↓serum glucose, serum insulin, HOMA-IR, GSSG, IL-6, oxidative stress<br>↑AMPK/GLUT4		C57BL/6 mice		*Oenanthe javanica, Hippophae rhamnoides, Ginkgo biloba* [61]
	Narcissoside	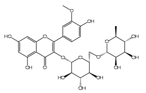	/	↓α-glucosidase		/		*Anoectochilus roxburghii, Peucedanum aucheri Boiss.* [62]

	Silibinin	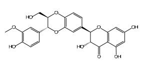	50μM; 140 μM	↓pdx1, ER stress (BiP, p-PERK, ATF6, XBP1), apoptosis (CHOP, Caspase12), autophagy (Beclin1, Atg5, LC3B), ROS, lipid peroxidation (↑GSH, GPX4, FSP1; ↓MDA, COX-2), ferroptosis ↑cell viability, insulin, neurod1, mafa, GLUT2, GLUT4, PINK1/parkin	RINm5F cells; GLUTag cells; INS-1 cells			[60,126]
Flavonols	Kaempferol	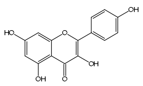	10^{-8}-10^{-6} M; 0.1, 1, 10 μmol/L; 50 mg/kg	↓body weight gain, FBG, HbAc1, serum glucose, IR, lipid accumulation, liver injury, α-glucosidase, α-amylase TC, TG, HDL ↑glucose tolerance, glucose uptake, insulin, AMPK/GLUT4, PI3K/AKT/GLUT4, Sirt1/AMPK, hexokinase *Desulfovibrio, butyricimonas*	HepG2 cells; human skeletal muscle cells	C57BL/6J mice; C57BLKS/J db/db mice; Male Wistar rats	Molecular Docking	Coreopsis tinctoria, *Euphorbia peplus* [49,51-54]
	Myricetin	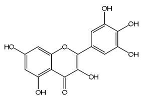	50, 200 mg/kg	↓serum glucose, insulin, pancreatic apoptosis (Bax, Bcl-2), SGLT1 ↑insulin receptor, GLUT4, G6Pase, PEPCK		Rats (HFD/STZ)		*Annona cherimola* Miller [55-56]
	Fisetin	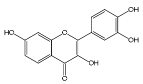	30 mg/kg	↓blood glucose, IR, AGEs, ROS, NF-Kb, inflammation ↑glucose tolerance	HK-2 cells	$LDLR^{-/-}$ mice		Cucumbers, onions, apples, strawberries [58]
	Astragalin	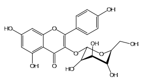	/	↓liver NF-kB p65, α-glucosidase, PEPCK, G6Pase, ↑hexokinase, liver PPARγ, glycogen synthase, PI3K-AKT-FOXO1/GSK-3β	HepG2 cells	Wistar rats	Molecular Docking	*Hypericum attenuatum, Euphorbia peplus* [49-50,139]
	Morin	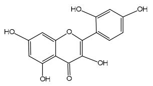	100 mg/kg	↓body weight gain, oxidative stress, inflammation (MCP-1, CCR2), TLR-4 ↑glucose tolerance, p-Akt, Nrf2		C57BL/6 mice, (HFD)		Moraceae family [59]
	Epimedin C	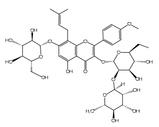	5, 10, 30 mg/kg	↓gluconeogenesis, FBG, HOMA-IR, lipid deposition, oxidative stress, hepatic injuries ↑glucose tolerance, hepatic glycogen, insulin		Specific pathogen free Kunming mice		*Epimedium* [57]

Flavanols	EGCG	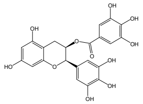	0.1, 1, 10 μM	↓PPT1B, LMW-PTP, α-amylase<br>↑p-insulin receptor, glucose uptake	HepG2 cells, C2C12 myoblasts			Indian green tea [64-65]
	(-)-Epicatechin-6	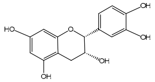	/	↑p-AMPK, glucose uptake		/		Australian *Acacia saligna* [63]
Flavanones	Naringenin	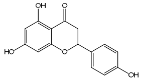	25 μM;<br>50, 100, 200, 300, 400 μM;<br>25, 50, 100 mg/kg;<br>50 mg/kg	↓islet β-cells apoptosis (Bax, caspase-3), inflammation, NF-κB, insulin, glucose, IR, ER stress (TRB3/Akt/FoxO1, PERK/eIF2α/ATF4/CHOP), TNFα, IL-1β<br>↑PI3K/AKT, glucose tolerance, GSIS, ERβ, p-p65, FGF21, ERα, p-Akt, p-FoxO1, Bcl2, IκBα	NRK 52E cells;<br>HepG2 cellss	C57BL/6J mice;<br>Wistar rats;<br>Sprague-Dawley rats		Citrus fruits (grapefruits, oranges) [73-76]
	Hesperetin	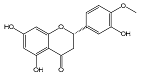	20 mg/kg	↓α-amylase, blood glucose, oxidative stress, inflammation, G6Pase, pancreatic damage<br>↑glucose tolerance, hepatic glycogen, insulin secretion, pancreatic cells		Wistar rats		[69-70]
	Hesperidin	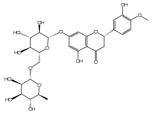	20, 50 μM	↓α-glucosidase			Molecular docking	Citrus fruits (orange, lemon, grapefruit) [72]
	Neoeriocitrin	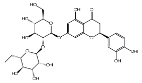	0.25, 0.5, 1μM	↓pancreatic apoptosis, ROS, lipid peroxidation<br>↑GSIS, antioxidant enzyme		/		[71]
	Pinocembrin	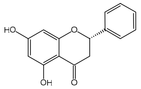	10, 50, 100 μM	↓lipid accumulation, IR<br>↑glucose uptake	HepG2 cells			Propolis [68]

Flavanones	Eriocitrin	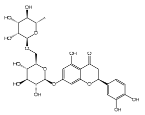	/	↓FBG, Bacteroidetes, Proteobacteria, *Blautia*, *Prevotella*, growth rate of Firmicutes and Lachnospiraceae<br>↑GLP-1, Actinobacteria, *Ruminococcaceae*		/		Lemon [108]
	Nigragenon O	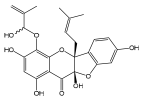	10, 30 μmol/L	↓glucose level, PPARγ<br>↑glucose uptake, adiponectin	3T3-L1 cells			*Mours nigra* [77]
	Bavachin	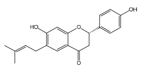	30 mg/kg	↓blood glucose, G6Pase<br>↑insulin sensitivity, p-AKT, p-GSK-3β, body weight	primary mouse hepatocytes, Huh7 cells, L02 cells, AML12 cells	C57BL/6 J mice (HFD)		*Psoralea corylifolia* L. [67]
Flavanonols	Dihydromyricetin (DHM)	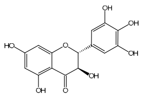	50, 100, 200 mg/kg; 250 mg/kg; 10 μM;	↓IR, hepatic steatosis, FBG, TG, HbA1c<br>↑hepatic autophagy (AMPK/PGC-1α, PPARα), FINS	HepG2 cells	Male C57BL/6 mice; SD rats;		*Ampelopsis grossedentata* [78-79]
	Sanggenon C	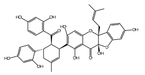	1, 5, 10, 15, 20 μM	↓oxidative stress, lipid accumulation, IR, ROS<br>↑glucose uptake, p-AMPK, IRS/AKT/FOXO1	HepG2 cells			*Cortex Mori* [80]
Biflavonoids	Involvenflavones	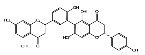	/	↑glucose consumption, GCK, ADCYs	HepG2 cells			*Selaginella tamariscina* Spring [81]
	Amentoflavone	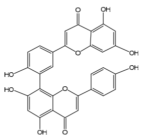	/	↓cell apoptosis, oxidative stress (↓ ROS, MDA, LDH; SOD, GSH, GSH-Px), IR, hIAPP, α-glucosidase<br>↑cell viability, glucose uptake, GLUT4, insulin secretion, AMPK/Nrf2	INS-1 cells; Rat H9C2 cardiomyocytes		Molecular docking, Molecular dynamics simulation	*Aletris Spicata*, *Selaginella Tamariscina* [82-84]

Biflavonoids	bilobetin	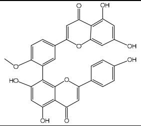	/	↓IR, hIAPP<br>↑insulin secretion	INS-1 cells			[83]
	hinokiflavone	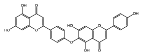	/	↓α-glucosidase			Molecular docking, Molecular dynamics simulation	[84]
Chalcones	Licochalcone A	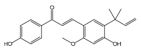	5, 10 mg/kg	↓mTORC1, gluconeogenesis, G6Pase, PEPCK<br>↑AMPK, hepatic glycogenesis, glucose tolerance, glucose uptake		C57BL/6J mice		Licorice [87]
	Neohesperidin dihydrochalcone	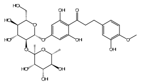	0.5 mg/mL	↓inflammation, liver lipid accumulation, FBG, serum glucose, IR, TNF-α, IL-6, G6Pase, PEPCK<br>↑GCK, PI3K/AKT		cloning diabetic zebrafish		[88]
	Nothofagin	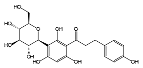	/	↓AGEs		/		*Aspalathus linearis* (Burman f.) R. Dahlgren [89]
	Phlorizin	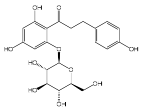	/	↓gluconeogenesis, oxidative stress, lipid accumulation<br>↑glucose consumption, glucose uptake, glycogen synthesis, AMPK/PI3K/AKT	HepG2 cells			*Lithocarpus litseifolius* (Hance) Chun [90]
Anthocyanidins	Cyanidin	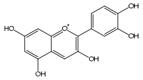	/	↓glucokinase		/		[96]

	Cyanidin-3-O-glucoside	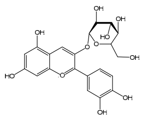	12.5, 25, 50 μM;<br>150 mg/kg	↓pancreatic β cell apoptosis (BAX, cleaved caspase-3), blood glucose, α-glucosidase, ER stress (PERK/eif2α/CHOP), inflammation (IL-1β), oxidative stress, IR, PTP1B<br>Firmicutes, Firmicutes/Bacteroidetes, *Burkholderiaceae*<br>↑GSIS, GLP-1, GLUT2, glycogen synthesis, glucose consumption, SCFAs, bile acids, insulin sensitivity<br>Bacteroidetes, *Oxalobacteraceae*, *Oxalobacter*	INS-1E cells; HepG2 and L02 cells	C57BL/J6 mice;<br>db/db mice	Molecular docking	Blueberry, red bayberry [91-94,96]
Anthocyanidins	Cyanidin-3-O-β-glucoside		/	↓bodyweight gain, hepatic lipogenesis, FBG<br>↑glucose tolerance, insulin sensitivity		Male C57BL6J mice		*Lonicera caerulea* L. [95]
	Delphinidin	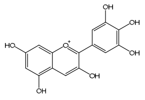	/	↑glucokinase		/		[96]
	Delphinidin-3-*O*-galactoside	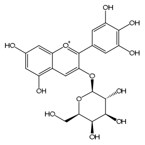	50, 100 μM	↓free radicals (ABTS⁺, DPPH), oxygen radical, α-glucosidase, AGEs, inflammation	RAW264.7 cells;<br>HepG2 cells		Molecular docking	Rabbiteye blueberry (*Vaccinium virgatum*) [97-98]
	Delphinidin-3-*O*-glucoside	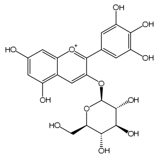	50, 100 μM	↓free radicals (ABTS⁺, DPPH), oxygen radical, α-glucosidase, AGEs, inflammation, lipid accumulation<br>↑glucose uptake	RAW264.7 cells;<br>HepG2 cells		Molecular docking	Rabbiteye blueberry (*Vaccinium virgatum*) [97-98]
Anthocyanidins	Malvidin-3-arabinoside	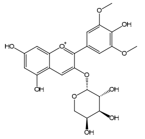	/	↓AGEs, inflammation	RAW264.7 cells			Rabbiteye blueberry (*Vaccinium virgatum*) [98]
	Malvidin-3-*O*-galactoside	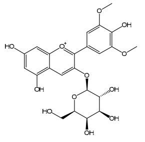	/	↓free radicals (ABTS⁺, DPPH), oxygen radical, α-glucosidase, TC, TG<br>↑glucose uptake	HepG2 cells			Rabbiteye blueberry (*Vaccinium virgatum*) [97]

Homoisoflavonoid	HM-chromanone	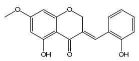	10, 30, 50 mg/kg; 20, 40 μM	↓blood glucose, IR, inflammation, p-IRS-1, gluconeogenic enzymes, p-IRS-1ser^{307}, PEPCK, G6pase ↑IRS-PI3K-AKT-GSK-3β/FOXO1, GLUT4, glycogen synthase	HepG2 cells	C57BL/6 J mice		*Portulaca oleracea* L. [99-101]
